# Design of metamagnetic layered cobalt(II) coordination polymers with controlled magnetic phase transitions

**DOI:** 10.3389/fchem.2026.1894406

**Published:** 2026-07-22

**Authors:** Miguel Cortijo, Isabel Coloma, Santiago Herrero, Reyes Jiménez-Aparicio, Emilio Matesanz

**Affiliations:** 1 Grupo MatMoPol, Departamento de Química Inorgánica, Facultad de Ciencias Químicas, Universidad Complutense de Madrid, Ciudad Universitaria, Madrid, Spain; 2 Université de Bordeaux, CNRS, Bordeaux INP, ICMCB, UMR 5026, Pessac, France; 3 Instituto de Tecnología del Conocimiento, Universidad Complutense de Madrid, Campus de Somosaguas, Madrid, Spain; 4 Unidad de Difracción de Rayos X, Centro de Asistencia a La Investigación de Técnicas Químicas, Universidad Complutense de Madrid, Ciudad Universitaria, Madrid, Spain

**Keywords:** 2D coordination polymers, magnetic phase diagrams, materials design, metamagnetism, quasi 1D magnetic behavior

## Abstract

Three coordination compounds of formula [CoCl_2_(*N*,*N′*)]_n_ [*N*,*N*' = 4,4′-bipyridine (4,4′-bpy), (**Cobpy**); *trans*-4,4′-azopyridine (*t*-apy), (**Coapy**); and *trans*-1,2-bis(4-pyridyl)ethylene (*t*-bpee), (**Cobpee**)] have been prepared using solvothermal procedures. They have been characterized by different techniques, including the *ab initio* crystal structure determination of **Cobpee**. The structures of **Cobpy** and **Cobpee** are formed by octahedral *tran*s-CoCl_4_N_2_ nodes, which construct rectangular grid layers that are stacked in an ABAB pattern. The three complexes show a similar metamagnetic behavior, which suggests that **Coapy** displays a layered structure equally built. The magnetic phase transitions of the compounds from an antiferromagnetic state to a paramagnetic-like or ferromagnetic-like state have been analyzed, and magnetic phase diagrams are provided. The use of *N*,*N′*-donor ligands with different lengths allows controlling the strength of the interchain interactions and the critical temperature (*T*_c_) and critical magnetic field (*H*_c_) of the metamagnetic phase transitions.

## 1 Introduction

The isolation of graphene and the discovery of its unique properties (Novoselov et al., 2004) boosted the interest of the scientific community in the study of other two-dimensional (2D) materials because of the potential applications that they display in several fields such as electronics (Zhan et al., 2019), quantum information science (Liu and Hersam, 2019), energy storage (Pomerantseva and Gogotsi, 2017), or hydrogen production (Ganguly et al., 2019). Most 2D materials reported to date are all-inorganic compounds that have stacked structures with strong bonding within each layer and weak van der Waals interactions between sheets, which is a prerequisite for the isolation of one-atom-thick materials by top-down techniques (Novoselov et al., 2016). Nonetheless, coordination chemistry has proven to be a powerful tool to prepare infinite 2D assemblies constructed by metal ions and bridging organic ligands covalently bonded (Li et al., 2019; Lu et al., 2018; Qin et al., 2022). This approach, encompassed in reticular chemistry, offers almost endless possibilities to design new materials with tunable properties by proper selection of the building blocks (Tran et al., 2019; Yaghi et al., 2003).

Several coordination-based 2D materials have been reported to date, highlighting the versatility of this strategy. For example, nickel bis(dithiolene) complexes (Kambe et al., 2013) and other layered compounds, such as [Ni_3_(2,3,6,7,10,11-hexaiminotriphenylene)_2_]_n_ (Sheberla et al., 2014), [Cu_3_(C_6_S_6_)]_n_ (Huang et al., 2015), or [M_3_ (hexaiminobenzene)_2_]_n_ (M = Ni, Cu) (Dou et al., 2017), exhibit high electrical conductivity that arises from a strong *π*-*d* conjugation of the orbitals of the ligand and metal ions. Layered coordination polymers of general formula [Fe(bimX)_2_]_n_ (HbimX = benzimidazole functionalized in the 5-position with X = H, Cl, Br, CH_3_ or NH_2_) are robust enough to be mechanically exfoliated by the scotch-tape method and are great candidates to study magnetism in the 2D limit (López-Cabrelles et al., 2018). Hybrid materials such as [BEDT-TTF]_3_[MnCr(C_2_O_4_)_3_] (BEDT = bis(ethylenedithio)tetrathiafulvalene), prepared by the combination of two functional sub-lattices, show both electrical conductivity and magnet properties (Coronado et al., 2000). The layered coordination compound [CrCl_2_(pyz)_2_]_n_ (pyz = pyrazine) shows ferrimagnetic ordering bellow 55 K and electrical conductivity induced by the redox activity of the pyrazine ligand and the presence of a reducing paramagnetic 3d metal (Pedersen et al., 2018). Theoretical calculations proved that a monolayer of this material is dynamically and thermally stable (Xie et al., 2020). In addition, the chemical reduction of [CrCl_2_(pyz)_2_]_n_ and [Cr(OSO_2_CH_3_)_2_(pyz)_2_]_n_ affords magnets with *T*_c_ up to 515 K and 500 Oersted *H*_c_ at room temperature (Perlepe et al., 2020). More recently, a molecular strategy for the development of 1D materials embedded in 2D coordination polymers has been explored using crystals of [FeX(pzX)(4,4′-bpy)] (pz = pyrazole; X = Cl, F) that show highly anisotropic optical properties (Mazarakioti et al., 2026).

**SCHEME 1:**
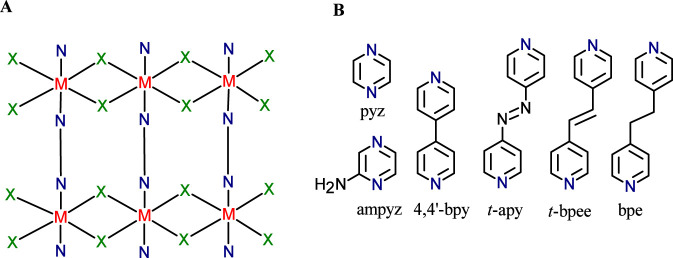
**(A)** Schematic representation of a layer of a [MX_2_(*N*,*N′*)]_n_ compound where M is a metal (II) ion, X is a halide or pseudohalide and *N*,*N′* is one of the ligands shown in **(B)** pyrazine (pyz), 2-aminopyrazine (ampyz), 4,4′-bipyridine (4,4′-bpy), *trans*-4,4′-azopyridine (*t*-apy), *trans*-1,2-bis(4-pyridyl)ethylene (*t*-bpee) or 1,2-bis(4-pyridyl)ethane (bpe).

Simple grid-like layers like that shown in Scheme 1, offer an attractive platform for exploring the coexistence of magnetic interactions within a single material. Several related layered compounds can be prepared by proper selection of paramagnetic transition metal(II) ions providing an octahedral environment, halide or pseudohalide ligands (X) and ditopic *N*,*N*′-donor ligands such as pyrazine (pyz), substituted pyrazines, 4,4′-bipyridine (4,4′-bpy), *trans*-4,4′-azopyridine (*t*-apy), *trans*-1,2-bis(4-pyridyl)ethylene (*t*-bpee), 1,2-bis(4-pyridyl)ethane (bpe) or pyrimidine (pym) (Haynes et al., 1986; Masciocchi et al., 1996; Manson et al., 1999; Lawandy et al., 1999; Hao et al., 2000; Feyerherm et al., 2002; Feyerherm et al., 2004; Ghosh et al., 2005; Danilovic et al., 2010; Wöhlert et al., 2011; Boonmak et al., 2011; Wöhlert et al., 2012; Karthikeyan et al., 2012; Wöhlert et al., 2013a; Wöhlert et al., 2013b; Cortijo et al., 2013a; Lapidus et al., 2013; De Campos et al., 2014; Wöhlert et al., 2014; Perlepe et al., 2022; Chen et al., 2023; Pitcairn et al., 2023; Vaidya et al., 2024; Pitcairn et al., 2024; Meng et al., 2026). Moreover, additional functionalities can be introduced in these systems or related ones using π-conjugated ligands with potential redox activity (Jurss et al., 2015; Karthikeyan et al., 2012; Pedersen et al., 2018).

Magnetic studies carried out for [MCl_2_(4,4′-bpy)]_n_ (M = Fe, Co, Ni, Co/Ni) complexes showed the existence of antiferromagnetic ordering and metamagnetic transitions originated by the coexistence of ferro- and antiferromagnetic interactions (Lawandy et al., 1999). A low temperature neutron diffraction study of the Ni and Co derivatives proved that the metallic centers are ferromagnetically coupled in the [MCl_2_]_n_ chains whereas the nearest chains, which belongs to contiguous layers, are antiferromagnetically coupled (Feyerherm et al., 2002). Other reported [NiCl_2_(*N*,*N*′)]_n_(*N*,*N*' = pyz, 4,4′-bpy, *t*-apy, *t*-bpee, bpe) complexes display a metamagnetic behavior, which can be controlled by modifying the nature of the organic linkers (Cortijo et al., 2013a). Additionally, coexistence of metamagnetism and slow relaxation of the magnetization was observed in the layered coordination polymers [CoX_2_(t-bpee)]_n_ (X = NCS (Wöhlert et al., 2011; Wöhlert et al., 2014), NCSe (Wöhlert et al., 2012) and [Co(NCS)_2_(bpe)]_n_ (Wöhlert et al., 2014) formed by magnetically isolated chains.

Subtle variations in the ligand environment and the metal coordination geometry enable fine control over magnetic exchange interactions, ultimately governing the emergence of field-induced phase transitions such metamagnetic behavior. On the basis of the above-mentioned precedents, we have undertaken the synthesis of a series of [CoCl_2_(*N*,*N*′)]_n_ complexes featuring Co(II) ions to investigate the existence of metamagnetism. In addition, we aimed at modulating metamagnetic behavior by changing the length of the *N*,*N*′-organic linkers and, therefore, controlling the strength of the interchain magnetic interactions. In order to do so, we report a general procedure under solvothermal conditions to synthesize layered complexes of formula [CoCl_2_(*N*,*N*′)]_n_ [*N*,*N*' = 4,4′-bipyridine (4,4′-bpy), (**Cobpy**); *trans*-4,4′-azopyridine (*t*-apy), (**Coapy**); and *trans*-1,2-bis(4-pyridyl)ethylene (*t*-bpee) (**Cobpee**)] and provide a detailed study of the variation of the magnetic properties along the series and its correlation with their crystal structure.

## 2 Experimental

### 2.1 Materials and methods

Solvothermal syntheses were carried out in a Memmert Universal Oven UFE 400 using 25 mL Teflon-lined stainless-steel autoclaves. All reactants and solvents were purchased from commercial sources and used without further purification.

### 2.2 Solvothermal synthesis (ST)

The complexes were obtained by reaction of CoCl_2_⋅6H_2_O (0.29 g, 1.22 mmol) and the corresponding *N*,*N′*-donor ligand (4,4′-bpy, *t*-apy, or *t*-bpee) (1.22 mmol) in a mixture of ethanol and water (9 and 3 mL, respectively).

The reactants and solvents were sealed in a Teflon-lined stainless-steel autoclave and heated at 120 °C for 1 day. The obtained solid was filtered, washed with ethanol (2 × 5 mL) and diethyl ether (2 × 5 mL), and dried under vacuum. **Cobpy**: Yield: 78%. Anal. Found (calcd) for H_8_C_10_N_2_Cl_2_Co (286.027 g · mol^-1^): C, 41.88 (41.99); H, 2.77 (2.82); N, 9.92 (9.79). **Coapy**: Yield: 75%. Anal. Found (calcd) for H_8_C_10_N_4_Cl_2_Co·0.25H_2_O (318.545 g · mol^-1^): C, 37.54 (37.71); H, 2.49 (2.69); N, 17.63 (17.59). **Cobpee**: Yield: 87%. Anal. Found (calcd) for H_10_C_12_N_2_Cl_2_Co (312.066 g·mol^-1^): C, 46.02 (46.19); H, 3.21 (3.23); N, 9.08 (8.98).

### 2.3 Physical measurements

Elemental analyses were performed by the Microanalytical Services of the Universidad Complutense de Madrid. FTIR spectra were recorded with a Perkin-Elmer Spectrum 100 with a universal ATR accessory in the 4000−650 cm^−1^ range (see Supplementary Section S1). Thermogravimetric analyses were performed in an air atmosphere up to 900 °C, with a heating rate of 5 °C min^−1^ using a Perkin-Elmer Pyris 1 TGA instrument (Supplementary Section S2). X-ray powder diffraction scans were measured in reflection mode on flat pressed powder samples (see Results and Discussion section, and Supplementary Section S3). The measurements were carried on in a Panalytical B.V. X’Pert PRO diffractometer with Cu tube and secondary flat graphite monochromator for X’Celerator fast detector in the detection optics, scanning from 5 to 120° in 2*θ* and an equivalent time per step of 500 s.

The magnetic data were obtained with a Quantum Design MPMS-XL SQUID magnetometer using 6.40, 7.17, and 11.28 mg of **Cobpy**, **Coapy** and **Cobpee**, respectively (see Results and Discussion section, and Supplementary Section S4). The samples were introduced in a polypropylene bag with a small amount of paratone oil to prevent movement of crystallites caused by the high magnetic anisotropy of the complexes. All data were corrected for the diamagnetic contribution of the sample, the sample holder and the oil. The temperature-dependence of the magnetization was measured in different intervals of interest of the 300-1.8 K range operating under fixed applied *dc* fields between 12 and 10000 Oe. The field-dependence of the magnetization was measured in different intervals of applied *dc* fields in the range from −30000 to 70000 Oe operating at fixed temperatures between 1.8 and 10 K. The *ac* susceptibility experiments were performed at different frequencies between 1 to 1500 Hz with *ac* field amplitude of 3 Oe in zero *dc* field.

## 3 Results and discussion

### 3.1 Synthetic aspects

The three compounds have been prepared by conventional solvothermal syntheses following similar procedures to those employed to prepare analogous Ni(II) compounds and other coordination polymers by our research group (Cortijo et al., 2013a; Cortijo et al., 2013b; Cortijo et al., 2014a; Cortijo et al., 2014b).

Although crystal growth is favored with solvothermal methods because the solubility and the diffusion rate of the reagents are enhanced in superheated solvents, all the attempts made in this work to obtain crystals suitable for single crystal X-ray diffraction were unsuccessful. Nevertheless, pure phases have been obtained, avoiding a possible mixture of polymorphs as was found for [CuCl_2_(4,4′-bpy)]_n_ (Masciocchi et al., 1996).

### 3.2 Thermogravimetric studies

The thermogravimetric curves of **Cobpy**, **Coapy** and **Cobpee**, measured in air atmosphere up to 900 °C with a heating rate of 5 °C min^−1^ (Supplementary Figures S2a‒S2c), show that the complexes are stable until at least 350 °C. The curves show a two-step weight loss pattern and the complexes decompose at high temperatures to form Co_3_O_4_. Supplementary Table S2a gives more detailed information about the observed weight loss and tentative assignments of the formed species. The identity of the oxide was confirmed by powder X-ray diffraction (Supplementary Figure S3a).

**Cobpy** is thermally stable up to 460 °C as shown in the corresponding thermogravimetric curve. At higher temperature, it starts to lose weight to form a product whose mass is compatible with [CoCl(4,4′-bpy)_0.5_]. Further heating leads to the formation of Co_3_O_4_ that begins at 535 °C. The curves for **Coapy** and **Cobpee** are similar: firstly, a weight loss compatible with the formation of [CoCl_2_(*N,N′*)_0.5_] is observed at 350 °C and 425 °C, respectively. The heating of these species gives rise to Co_3_O_4_ at 430 °C and 490 °C, respectively.

### 3.3 X-ray refinements

The structure of **Cobpy** was previously reported (Lawandy et al., 1999) as other structures of similar [MX_2_(4,4′-bpy)]_n_ compounds with different metal ions and/or X ligands [M = Mn (Chippindale et al., 2000), Fe (Lawandy et al., 1999), Ni (Cortijo et al., 2013a; Lawandy et al., 1999; Masciocchi et al., 1996), Co/Ni (Lawandy et al., 1999), Cu (Masciocchi et al., 1996), Zn (Chen et al., 2006; Hu and Englert, 2005; Saha et al., 2017), Cd (Biradha and Fujita, 2001; Chen et al., 2006; Chen et al., 2007; Hu et al., 2003; Niu et al., 2005; Zhu et al., 2005), Hg (Chen et al., 2006; Xie and Wu, 2007). To the best of our knowledge, there is no analogous crystal structure containing the *t*-apy ligand, and **Cobpee** and [NiCl_2_(*t*-bpee)]_n_ (Cortijo et al., 2013a) are the only two examples with the *t-*bpee ligand.

The powder pattern of **Cobpy** was identified with the already described structure corresponding to the CSD (Allen, 2002) entry QEJCOX (Lawandy et al., 1999), which crystallizes in the *Cmmm* space group. To verify this point, a Rietveld refinement (Rietveld, 1967; Rietveld, 1969) was performed against the experimental scan keeping fixed the atomic coordinates during the refinement. For this purpose, Fullprof Rietvled software was used (Rietveld, 1967; Rietveld, 1969; Rodríguez-Carvajal, 1993). Good agreement factors were found (Supplementary Figure S3b).

The compounds **Coapy** and **Cobpee** were not identified with any described crystalline phase, so a unit cell parameter determination was attempted by means of the different indexing methods implemented in Panalytical B.V. X’Pert HighScore software (Degen et al., 2014). The poor quality of the powder pattern of **Coapy** prevented a proper indexing (Supplementary Figure S3c), but the unit cell and space group determination of the compound **Cobpee** were successful. The cell parameters obtained for **Cobpee** were close to those of compound [NiCl_2_(bpe)]_n_ (bpe = 1,2-bis(4-pyridyl)ethane) (Cortijo et al., 2013a), sharing even the space group *P*2_1_/*a*. The ligands in these compounds are not identical, but they are similar enough to suggest the same structural arrangement.

**FIGURE 1:**
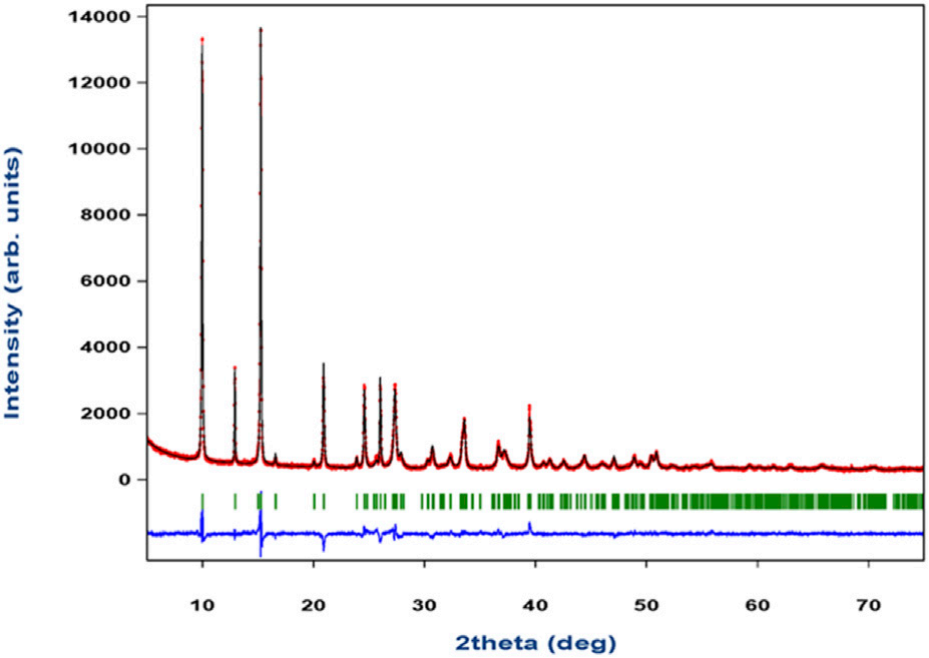
Rietveld refinement for **Cobpee**. Red dots represent experimental XRD scan whereas the black line represents the computed scan. Green ticks are the peak positions for the refined phase. Blue line shows the difference between the observed and calculated patterns.

A starting structural model was proposed for compound **Cobpee** considering chains of CoCl_2_, in the same disposition as those in the related Ni phase, separated by the *trans*-1,2-bis(4-pyridyl)ethylene ligand. Half the organic moiety included in the asymmetric unit was refined as a rigid body keeping unrefined the relative atomic positions of the starting model obtained from the WEHVIP CSD entry (Li et al., 2006). The Rietveld refinement was carried out considering anisotropic broadening of the peaks and only a *B* overall was refined. Only the portion of the diffractogram between 5 and 75° in 2*θ* was fitted, as beyond this angle no diffraction features could be appreciated. The proposed model probed to be good enough as shown in the results for this refinement included in Table 1 and Figure 1. Although bond distance for the C6-C6′double bond is lower than expected, this can be explained on the low resolution diffractogram available for this structural determination. The data can be obtained free of charge from the Cambridge Crystallographic Data Centre via www.ccdc.cam.ac.uk/data_request/cif.

**TABLE 1:** Refinement data obtained for **Cobpee**.

Space group	*P*2_1_/*a* (14)
*a* (Å)	11.7033 (3)
*b* (Å)	13.6879 (2)
*c* (Å)	3.64788 (7)
*Beta* (°)	97.276 (2)
*R*_p_	5.68
*R*_wp_	7.33
*R*_exp_	4.27
*Chi* ^2^	2.95
*R*_Bragg_	7.10
*R*_F_	4.61
*B* overall	2.6 (1)
CCDC Deposition #	1489200

### 3.4 Description of the structures

The most relevant bond distances and angles for **Cobpy** (Lawandy et al., 1999) and **Cobpee** are listed in Table 2. The Co(II) centers display an octahedral geometry with four chloride ligands and two nitrogen atoms of two *N*,*N′*-donor ligands in *trans* disposition. The octahedral are slightly distorted due to the differences in the Co-Cl and Co-N distances and the Cl-Co-Cl, Cl-Co-N, N-Co-N and Co-Cl-Co angles (Table 2).

**TABLE 2:** Summary of some relevant distances (Angstroms) and angles (deg) found in **Cobpy** and **Cobpee**.

Compound	Cobpy (Lawandy et al., 1999)	Cobpee
Co-Cl	2.487 (2)	2.506 (5)
Co-N	2.152 (9)	2.049 (7)
Cl-Co-Cl	86.98 (8) 93.11 (8)	84.6 (2) 95.4 (2)
Cl-Co-N	90	85.9(1), 94.1(1)
N-Co-N	180	180
Co-Cl-Co	93.11 (8)	95.4 (2)
Co···Co (*b* axis)	11.374 (2)	13.6879 (2)
Co···Co (*c* axis)	3.611 (1)	3.6479 (1)
Co···Co (Interlayer)	8.264 (1)	9.0045 (1)

**FIGURE 2:**
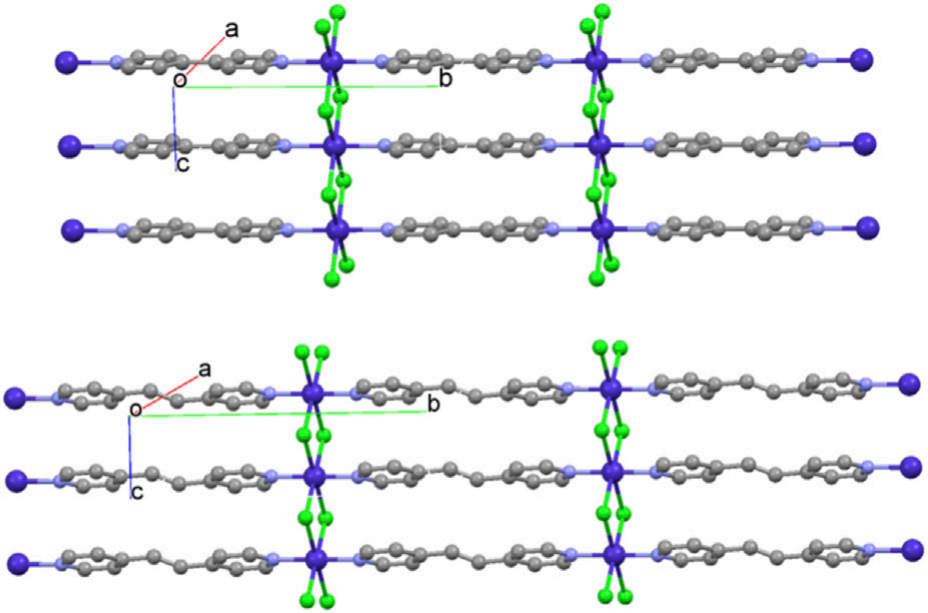
View of a layer of **Cobpy** (top) and **Cobpee** (bottom). Blue: Cobalt. Green: Chloride. Purple: Nitrogen. Grey: Carbon. Hydrogen atoms have been omitted for clarity. Structural data of **Cobpy** taken from Lawandy et al. (1999).

**Cobpy** and **Cobpee** display a rectangular grid layered structure in which the *N*,*N′*-donor ligands bridge Co(II) centers along the *b* axis while the chloride ions bridge Co(II) ions along the *c* axis (Figure 2). Therefore, the Co···Co distances along the *b* axis for **Cobpy** and **Cobpee** are very different given the length of the *N*,*N*′ organic linkers, while the Co···Co distances along the *c* axis are similar. As can be seen in Figure 2, π-π interactions are established between aromatic rings of the same layer.

**FIGURE 3:**
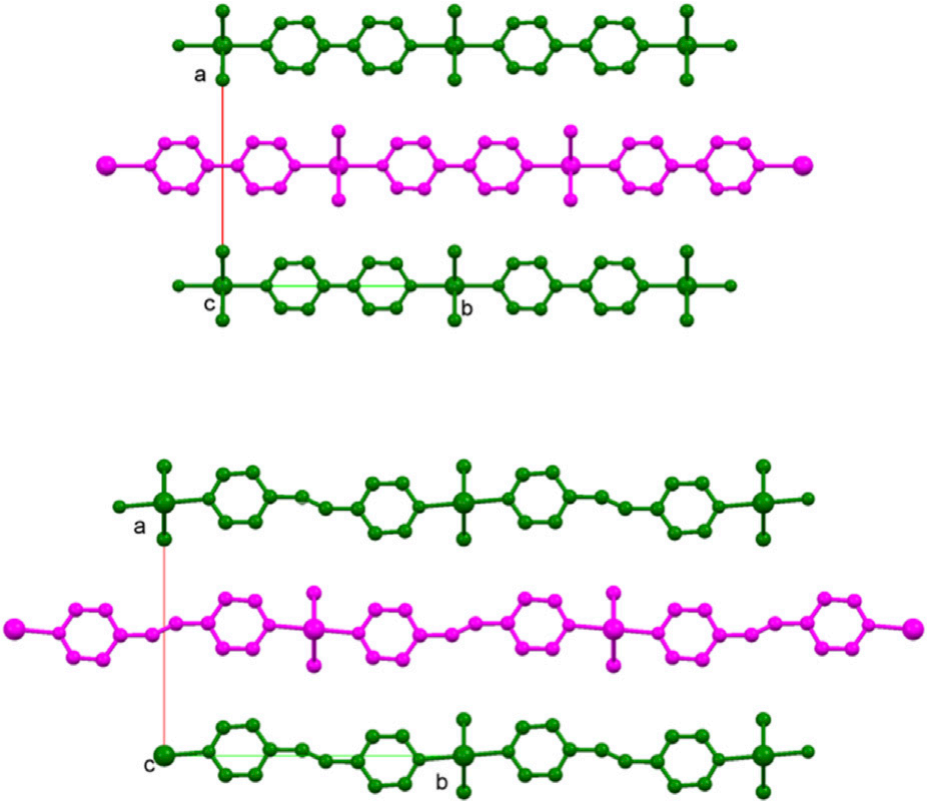
View along *c* of the structure of **Cobpy** (top) and **Cobpee** (bottom) with different layers depicted in green and magenta. Structural data of **Cobpy** taken from Lawandy et al. (1999).

The different layers are stacked along the *a* axis in an ABAB pattern as shown in Figure 3. The shortest interlayer Co··Co distance is 8.264 (1) Å for **Cobpy** and 9.0045 (1) Å for **Cobpee**. Note that this distance is also larger for the compound with the larger *N*,*N′-*donor ligand, [CoCl_2_(*t*-bpee)]_n_ (**Cobpee**), and that both distances are notably shorter than their respective Co···Co distances along the *b* axis.

### 3.5 Magnetic properties

**FIGURE 4:**
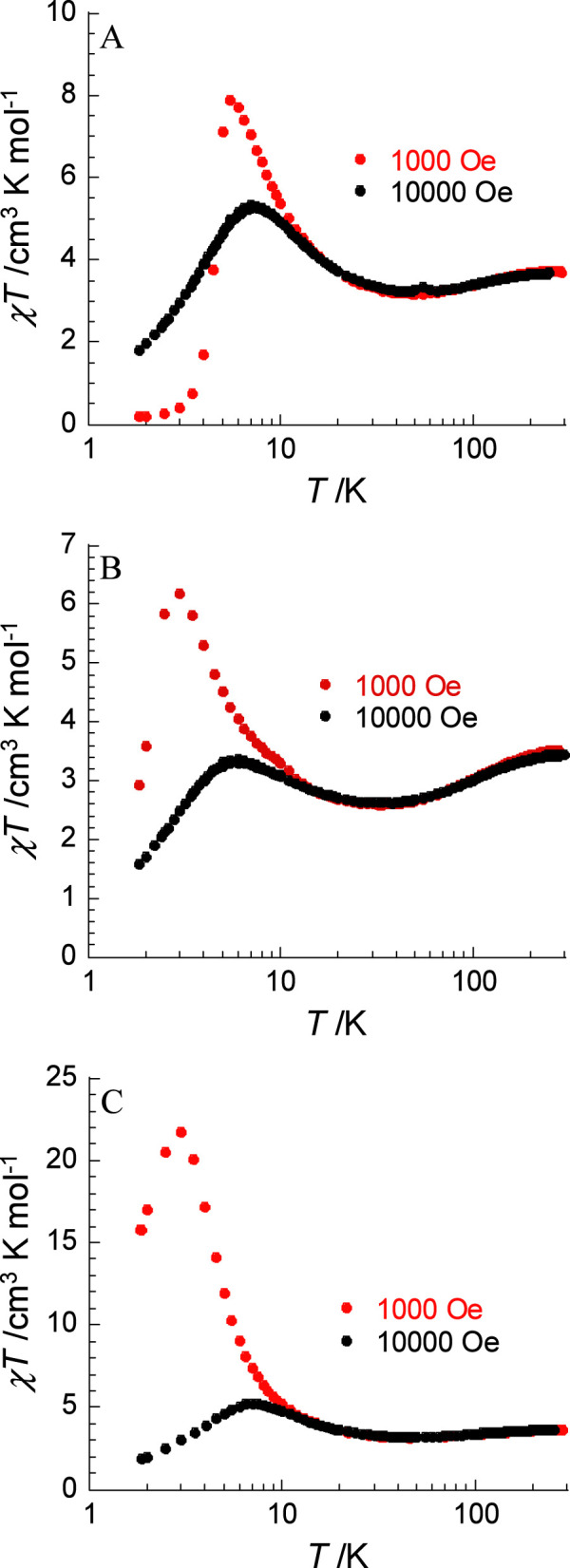
Temperature dependence of the molar magnetic susceptibility-temperature product, *χT*, for **(A) Cobpy**, **(B) Coapy** and **(C) Cobpee** at 1000 and 10000 Oe. The *x*-axis is in logarithmic scale.

The magnetization of **Cobpy**, **Coapy** and **Cobpee** was measured between 300 K and 1.8 K under applied *dc* fields of 1000 and 10000 Oe. The molar magnetic susceptibility-temperature product, *χT*, at these two different magnetic fields are largely superimposed from room temperature to approximately 15 K (Figure 4; Supplementary Figures S4a-S4c).

The *χT* values at room temperature are in the 3.72−3.51 cm^3^ K mol^−1^ range. These values are much larger than the calculated spin-only value for a *S* = 3/2 with *g* = 2 (1.875 cm^3^ K mol^−1^), as usual in octahedral high spin Co(II) complexes with significant orbital contribution. On cooling, a steadily decrease of the *χT* value, ascribed to spin-orbit coupling, until to *ca* 40 K for **Cobpy** and **Cobpee** and 30 K for **Coapy** is observed. Further cooling leads to field-dependent changes in the curves originated by magnetic interactions between Co(II) ions. Lowering the temperature under an applied field of 1000 Oe results in an increase of *χT*, ascribed to ferromagnetic interactions, until a maximum is reached at 5.5 K for **Cobpy** and at 3.0 K for both **Coapy** and **Cobpee**. Similarly, when the applied field is 10000 Oe the maximum is observed at 7.0 K for both **Cobpy** and **Cobpee** and at 6.0 K for **Coapy**. Lowering the temperature below the maxima (at 1000 or 10000 Oe) results in a sharp decrease of *χT* ascribed to dominant antiferromagnetic interactions.

**FIGURE 5:**
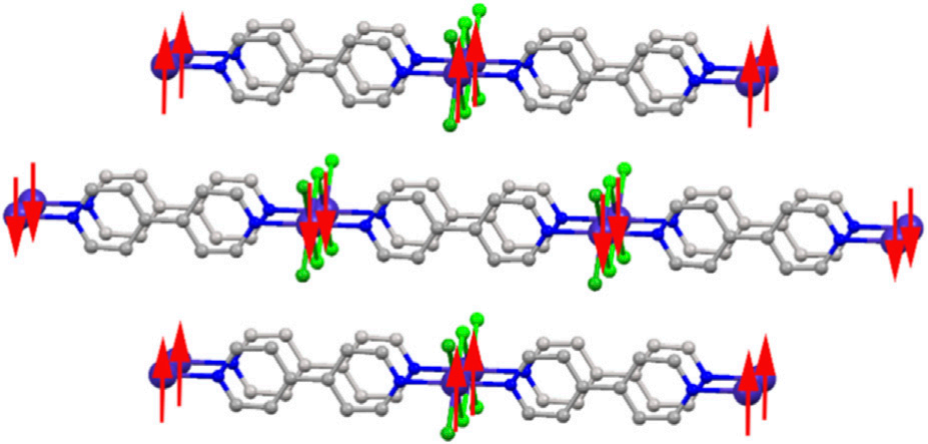
Spin arrangement in the antiferromagnetic ground state of **Cobpy**.

The data obtained for this family of compounds are in agreement with previous magnetic studies of **Cobpy** including a neutron diffraction study of [CoCl_2_(4,4′-bpy-*d*_8_)]_n_ carried out at 1.5 and 6 K (Feyerherm et al., 2002) that reveal that the overall spin arrangement of **Cobpy** is antiferromagnetic with a *T*_c_ of 5.0 K. The neutron diffraction experiments indicate that in the ground state i) the spins are arranged ferromagnetically (*J*) along the [CoCl_2_]_n_ chains, ii) there are interlaminar antiferromagnetic interactions (*J′*) and iii) the ordered spins are oriented along the 4,4′-bpy-*d*_8_ ligands (Figure 5).

In this work, the strength of the ferromagnetic interactions (*J*) along the [CoCl_2_]_n_ chains of **Cobpy**, **Coapy** and **Cobpee** have been estimated through the temperature dependence of the correlation length (ξ), which quantify the length of correlated spins in one-dimensional systems. At low temperatures (within the Ising limit, $\left|D/J\right|\;>\;4/3$ ), *x'T* increases exponentially in zero *dc* field with lowering temperatures following this expression: $x^{\prime }T\approx C_{eff}\;\exp\left({\Delta_{\xi }/k}_{B}T\right)$ (where *χ′* is the in-phase *ac* molar susceptibility, *C*_eff_ is the Curie Constant and ${\Delta_{\xi }/k}_{B}$ represents the energy to create a domain wall in the chain and it is equal to $4JS^{2}$ ) (Coulon et al., 2006). Consequently, magnetic measurements with zero *dc* field at 1 Hz frequency were carried out. The ln(*x*'*T) vs* 1/*T* plots obtained from these measurements are shown in Supplementary Figures S4d-S4f. The ${\Delta_{\xi }/k}_{B}$ values obtained from the slope of the linear regions of these plots are shown in the Supplementary Material and lead to *J*/*k*_B_ values of 0.78, 0.36 and 1.02 K for **Cobpy**, **Coapy**, and **Cobpee**, respectively.

The ln(*x'T) vs* 1/*T* plots deviate from linearity at very low temperatures because of the interchain antiferromagnetic interactions (*J′*). For this reason, the temperature and field dependence of the magnetization of **Cobpy**, **Coapy**, and **Cobpee** have been thoroughly studied below 10 K (see below). These measurements have allowed us to prove the existence of an antiferromagnetic ground state in **Coapy** and **Cobpee** and compare it with that of **Cobpy** (Feyerherm et al., 2002). An antiferromagnetic ground state was also found for related compounds with other metal centers: [NiCl_2_(4,4′-bpy)], [NiCl_2_(*t-*apy)] and [NiCl_2_(*t-*bpee)] and [FeCl_2_(4,4′-bpy)] (Cortijo et al., 2013a; Lawandy et al., 1999).

**FIGURE 6:**
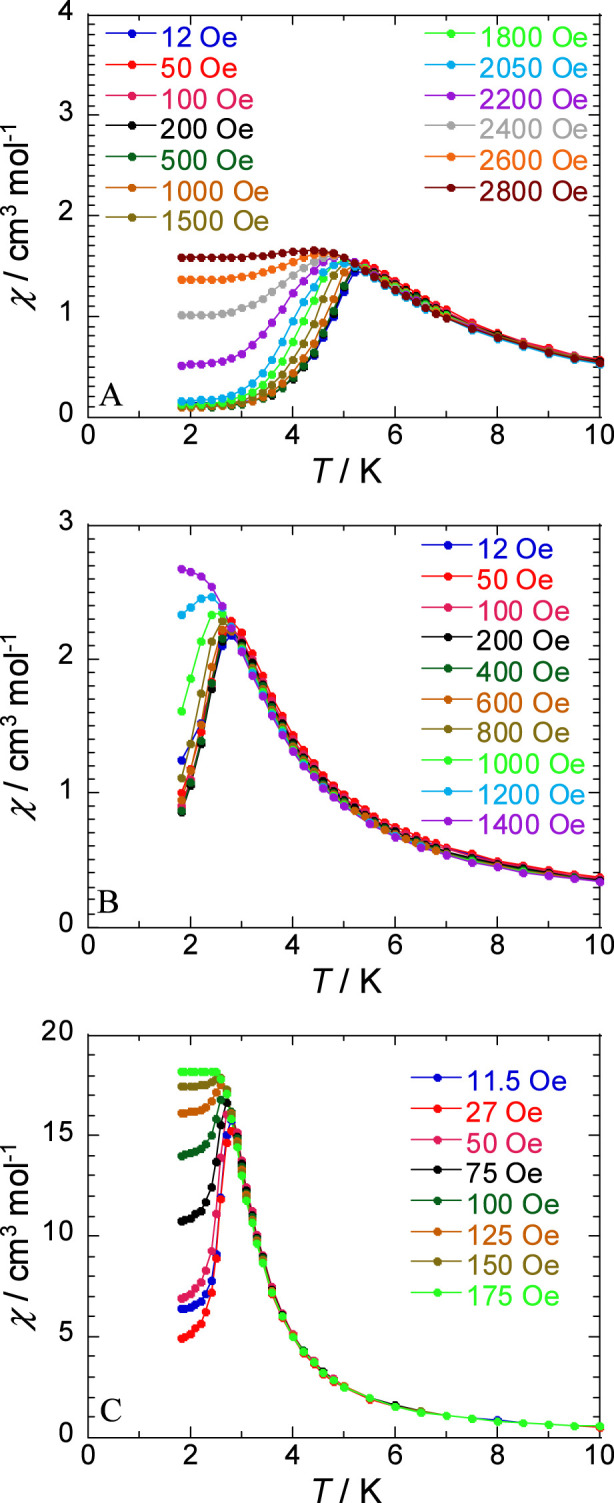
Temperature dependence of the molar magnetic susceptibility, *χ*, for **(A) Cobpy** at magnetic fields ranging from 12 to 2800 Oe, for **(B) Coapy** at magnetic fields ranging from 12 to 1400 Oe and **(C) Cobpee** at magnetic fields ranging from 11.5 to 175 Oe. Solid lines are just a guide for the eye.

The *χ vs T* data of **Cobpy** at 12 Oe shows a maximum at 5.2 K that is shifted to lower temperatures when the magnetic field is increased. This effect is observed under applied fields up to 2600 Oe and vanishes under higher applied magnetic fields (Figure 6A). A similar behavior is observed in the *χ vs T* data of **Coapy** and **Cobpee**. A maximum in the *χ vs T* data is observed at 2.8 K for **Coapy** and **Cobpee** under an applied magnetic field of 12 and 11.5 Oe, respectively. These maxima are shifted to lower temperatures with increasing magnetic fields and are not observed under a magnetic field of 1400 Oe for **Coapy** and 175 Oe for **Cobpee** (Figures 6B,C).

**FIGURE 7:**
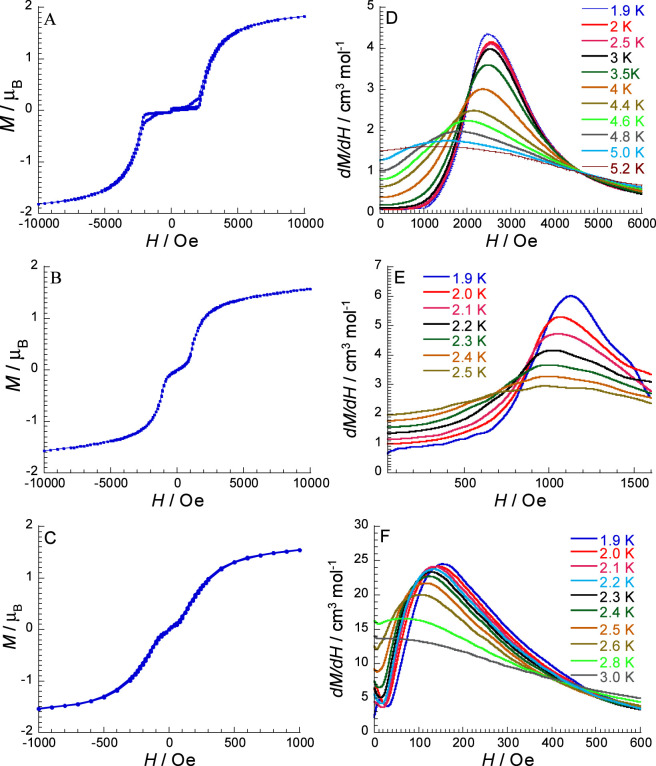
Field dependence of the magnetization at 1.9 K for **(A) Cobpy**, and **(B) Coapy** from −10000 to 10000 Oe and for **(C) Cobpee** from −1000 to 1000 Oe. d*M/*d*H vs H* plots for temperatures ranging from 1.9 to 5.2 K for **(D) Cobpy**, ranging from 1.9 to 2.5 K for **(E) Coapy** and ranging from 1.9 to 3.0 K for **(F) Cobpee**.

*M vs H* measurements at 1.9 K show the typical shape of a metamagnetic behavior with a *H*_c_ that separates an antiferromagnetic ground state from a paramagnetic-like or ferromagnetic-like state for **Cobpy**, **Coapy**, and **Cobpee** (Figures 7A–C; Supplementary Figures S4g-S4i). The lack of saturation observed at high field in the measurements is probably caused by some orientation effect of the microcrystalline powder. In addition, the *M(H)* plot of **Cobpy** shows no reversibility from *ca*. −2000 to 2000 Oe but neither remanent magnetization nor coercivity are observed.

*H*_c_ values have been estimated from the maxima of the d*M/*d*H vs H* plots (Figures 7D–F). At 1.9 K, these values are 2555, 1130, and 155 Oe for **Cobpy**, **Coapy**, and **Cobpee**, respectively. A shift of the *H*_c_ to lower values is observed at higher temperatures. The maximum is flattened at temperatures higher than 5.0 K for **Cobpy**, 2.4 K for **Coapy**, and 2.6 K for **Cobpee**. More information including *M(H)* plots at temperatures ranging from 1.9 to 10 K can be found in Supplementary Figures S4j-S4l. The analogous compound [NiCl_2_(4,4′-bpy)] also presents metamagnetic behavior but with much higher *H*_c_ values at 2 K (*H*_c1_ = 6.5 KOe and *H*_c2_ = 12 KOe) while [FeCl_2_(4,4′-bpy)] presents a lower *H*_c_ value of 3.5 KOe (Cortijo et al., 2013a; Lawandy et al., 1999).

**FIGURE 8:**
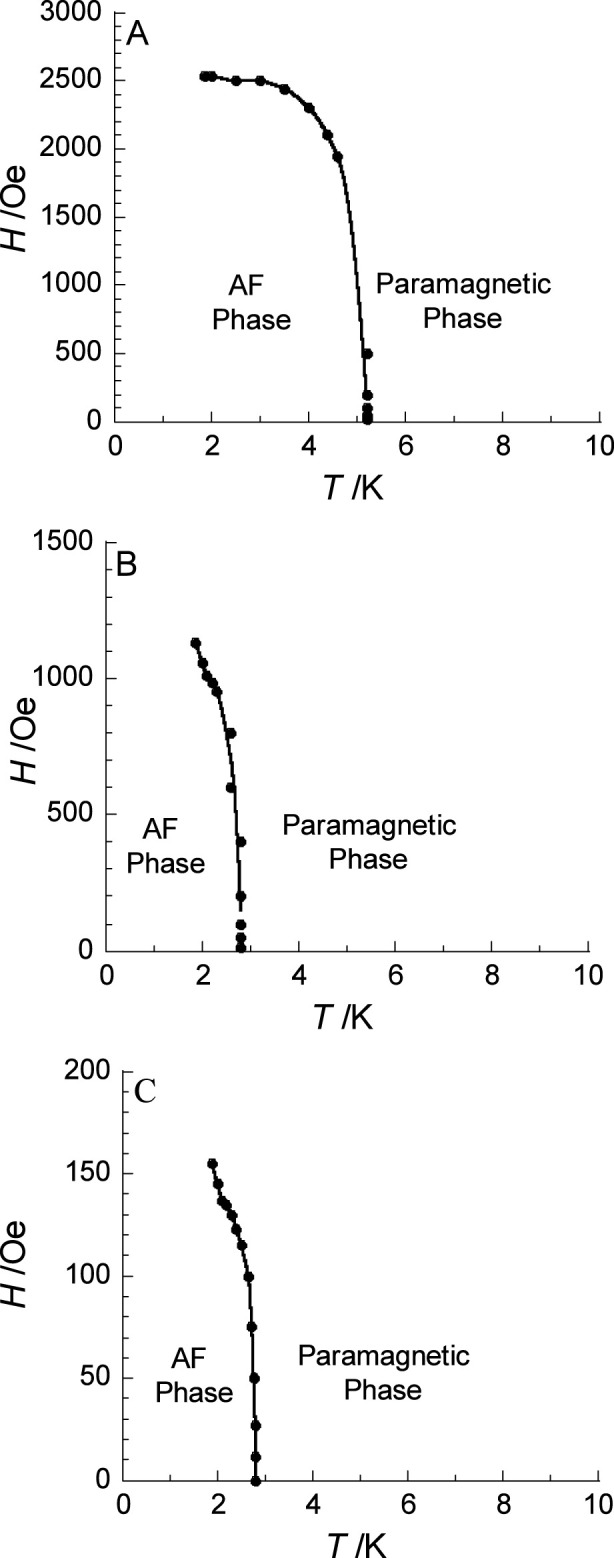
Magnetic (*T, H*) phase diagrams for **(A) Cobpy**, **(B) Coapy**, and **(C) Cobpee**.

Magnetic (*T, H*) phase diagrams built with the *T*_c_ data estimated at different applied fields and *H*_c_ estimated at different temperatures are shown in Figure 8. The interlaminar antiferromagnetic interactions (*J′*) can be calculated with the following expression obtained from equalizing the exchange and the Zeeman energies: $2\cdot z\cdot \left|J^{\prime }\right|\cdot S^{2}=g\cdot \mu_{B}\cdot H_{c}\left(0\;K\right)\cdot S$ (*z* = 4 as can be seen in Figure 3). Considering *H*_c_(0) values of 2555, 1130, and 155 Oe, the interlaminar antiferromagnetic interactions have been estimated to be *J'*/k_B_ ≈ −29, −13, and −2 mK for **Cobpy**, **Coapy**, and **Cobpee**, respectively. Note that the *H*_c_(0) values employed for **Coapy** and **Cobpee** are probably slightly underestimated as can be deduced by the shape of the corresponding phase diagrams.

From a magnetic point of view, these compounds can be considered as quasi-one-dimensional systems because of their high $\left|J\left(intrachain\right)/J^{´}\left(interchain\right)\right|$ value, which is especially high in the case of **Cobpee** ( $\left|J/J^{´}\right|=$ 510). The quasi-one-dimensional magnetic character of **Cobpy**, **Coapy**, and **Cobpee** and the coexistence of metamagnetism and Single-Chain Magnet (SCM) behavior in related compounds such as [CoX_2_ (*t*-bpee)]_n_ (X = NCS (Wöhlert et al., 2011; Wöhlert et al., 2014), NCSe (Wöhlert et al., 2012) and [Co(NCS)_2_ (bpe)]_n_ (Wöhlert et al., 2014) prompted us to study the *ac* susceptibility of **Cobpy**, **Coapy**, and **Cobpee**. However, no significant out-of-phase *ac* signals were detected above 1.8 K confirming the absence of slow relaxation of the magnetization in **Cobpy**, **Coapy**, and **Cobpee** (Supplementary Figures S4m-S4o in the Supplementary Material).

The positive sign of the intrachain interactions (*J*) is ascribed to the fact that the chains contain cobalt(II) ions bridged by two chloride ligands making angles close to 90°, leading to orthogonal *d* (cobalt) and *p* (chloride) orbitals, as predicted by Anderson’s superexchange theory (Anderson, 1963). The magnetic one-dimensional character is enhanced with longer *N*,*N*′-donor ligands that lead to longer interchain and interlayer Co···Co distances (Table 2) and weaker antiferromagnetic interactions through space. This trend is clearly observed in the variation of the *T*_c_ and *H*_c_ values (Table 3).

**TABLE 3:** *T*_c_ and *H*_c_ values for **Cobpy**, **Coapy**, and **Cobpee** and related metamagnetic compounds.

Compound	*T*_c_/K	*H*_c_/Oe
**Cobpy**	5.2	2555
**Coapy**	2.8	1130
**Cobpee**	2.8	155
CoCl_2_·2H_2_O (Weitzel and Schneider, 1974)	17.3	31800^*b*^
[CoCl_2_(pyridine)_2_]_n_ Foner et al. (1978)	3.2	700^*a*^ 800,^*b*^ 1600^*b*^ 4200^*c*^
[CoCl_2_(thiazole)_2_] (James and Horvat, 2002)	11.0	Data not provided

^*a*^,^*b*^,^*c*^Direction of the applied magnetic field.

The *T*_c_ and *H*_c_ values obtained for **Cobpy**, **Coapy**, and **Cobpee** are lower than those of other related systems with a 1D structure (Table 3). For example, CoCl_2_⋅2H_2_O displays much higher *T*_c_ and *H*_c_ parameters given stronger intrachain antiferromagnetic interactions (Weitzel and Schneider, 1974). This compound exhibits a metamagnetic behavior that separates an antiferromagnetic state from a paramagnetic one (*T*_c_ of 17.3 K and a *H*_c_ of 31800 Oe applied along the *b* axis) and a triple point at 37500 Oe and 8.9 K in which antiferro-ferri and paramagnetic states meet. Another similar system, whose structure is formed by chains, is the [CoCl_2_(pyridine)_2_]_n_ complex (Foner et al., 1978), which displays a *T*_c_ of 3.2 K being lower than that of **Cobpy**, and slightly higher than that of **Coapy** and **Cobpee**. [CoCl_2_(pyridine)_2_]_n_ displays two metamagnetic transitions at 800 and 1600 Oe for applied fields along the *b* axis and a single transition at 700 Oe and 4200 Oe for applied fields along *a* axis and *c* axis, respectively. Another relevant example is [CoCl_2_(thiazole)_2_]_n_ (James and Horvat, 2002), which also displays metamagnetic behavior, with a *T*_c_ of 11 K, arising from intrachain ferromagnetic interactions and weak interchain antiferromagnetic interactions. In light of these results, the variation of the *N*,*N*′-donor bridging ligands allows the preparation of a family of layered materials featuring nearly magnetically isolated [CoCl_2_]_n_ chains which exhibit controlled metamagnetic behavior with lower *T*_c_ and *H*_c_ values than other related compounds formed by 1D structures. Finally, it is worth mentioning that the overall magnetic data and the variation of their properties along the series of the three compounds is a strong support of the assumption that the three compounds have the same type of structure.

## 4 Conclusion

The magnetic behavior of [CoCl_2_(*N*,*N′*)]_n_ coordination complexes with a layered structure, formed by rectangular grid sheets with octahedral *tran*s-CoCl_4_N_2_ nodes, can be modulated by proper selection of *N*,*N*′-donor ligands. The coexistence of ferromagnetic interactions (*J*) along the [CoCl_2_]_n_ direction and interchain antiferromagnetic interactions (*J′*) originates a metamagnetic behavior when 4,4′-bipyridine (4,4′-bpy), *trans*-4,4′-azopyridine (*t*-apy), or *trans*-1,2-bis(4-pyridyl)ethylene (*t*-bpee) are used as *N*,*N′* ligands affording **Cobpy**, **Coapy**, and **Cobpee** complexes, respectively. The different length of these ligands is used to modulate the strength of *J′* and control the values of *T*_c_ and *H*_c_. Although these complexes display an antiferromagnetic ground state, their high $\left|J\left(intrachain\right)/J^{´}\left(interchain\right)\right|$ estimated from the magnetic measurements indicate that [CoCl_2_]_n_ chains are almost magnetically isolated. The low values obtained for *T*_c_ and *H*_c_, especially in the case of **Cobpee**, indicate that the magnetic isolation of the chains is more effective than in other related metamagnetic complexes with 1D structure.

## Data Availability

The datasets presented in this study can be found in online repositories. The names of the repository/repositories and accession number(s) can be found in the article/Supplementary Material.
