# Supplementary material for: Design of metamagnetic layered cobalt(II) coordination polymers with controlled magnetic phase transitions
Source: Front Chem. 2026 Jul 22;14:1894406. doi: 10.3389/fchem.2026.1894406 (PMC13438203; doi:10.3389/fchem.2026.1894406)
Supplement: Supplementary file 1 [file DataSheet1.pdf]

Supplementary Material

Design of metamagnetic layered cobalt(II) coordination polymers with controlled magnetic phase transitions

Miguel Cortijo[1*], Isabel Coloma[2], Santiago Herrero[1,3*], Reyes Jiménez-Aparicio[1], Emilio Matesanz[4]

[1]Grupo MatMoPol, Departamento de Química Inorgánica, Facultad de Ciencias Químicas, Universidad Complutense de Madrid, Ciudad Universitaria, E-28040 Madrid, Spain.

[2]Université de Bordeaux, CNRS, Bordeaux INP, ICMCB, UMR 5026, F-33600 Pessac, France

[3]Instituto de Tecnología del Conocimiento, Universidad Complutense de Madrid, Campus de Somosaguas, E-28223 Pozuelo de Alarcón, Madrid, Spain.

[4]Unidad de Difracción de Rayos X, Centro de Asistencia a la Investigación de Técnicas Químicas, Universidad Complutense de Madrid, Ciudad Universitaria, E-28040, Madrid, Spain.

Correspondence:

Miguel Cortijo
miguelcortijomontes@ucm.es

Santiago Herrero
sherrero@ucm.es

Contents

S1. Infrared spectra

Figure S1a. IR spectrum of $[CoCl_2(N,N')]_n$ [*N*,*N*' = 4,4'-bipyridine (4,4'-bpy), (**Cobpy**).

Figure S1b. IR spectrum of $[CoCl_2(N,N')]_n$ [*N*,*N*' = *trans*-4,4'-azopyridine (*t*-apy), (**Coapy**).

Figure S1c. IR spectrum of $[CoCl_2(N,N')]_n$ [*N*,*N*' = *trans*-1,2-bis(4-pyridyl)ethylene (*t*-bpee), (**Cobpee**).

S2. Thermogravimetric analysis

Figure S2a. Thermogravimetric curve for **Cobpy** and tentative assignments of the formed species.

Figure S2b. Thermogravimetric curve for **Coapy** and tentative assignments of the formed species.

Figure S2c. Thermogravimetric curve for **Cobpee** and tentative assignments of the formed species.

Table S2a. Weight loss observed for **Cobpy**, **Coapy** and **Cobpee** in their thermogravimetric curves and tentative assignments of the formed species.

S3. Powder X ray diffraction of the compounds

Figure S3a. Powder X-ray diffraction data for Co_3O_4 obtained after the thermogravimetric measurement of **Cobpy** in air.

Figure S3b. Rietveld refinement plot for **Cobpy**.

Figure S3c. X-ray powder diffraction scan for **Coapy**.

S4. Magnetic measurements

Figure S4a. Temperature dependence of χT for **Cobpy** at 100, 1000 and 10000 Oe.

Figure S4b. Temperature dependence of χT for **Coapy** at 1000 and 10000 Oe.

Figure S4c. Temperature dependence of χT for **Cobpee** at 1000 and 10000 Oe.

Figure S4d. Ln $\chi' T$ *vs* $1/T$ plot of **Cobpy** in zero *dc* field at 1 Hz.

Figure S4e. Ln $\chi' T$ *vs* $1/T$ plot of **Coapy** in zero *dc* field at 1 Hz.

Figure S4f. Ln $\chi' T$ *vs* $1/T$ plot of **Cobpee** in zero *dc* field at 1 Hz.

Figure S4g. Field dependence of the magnetization for **Cobpy** at 1.9 K from -30000 Oe to 70000 Oe.

Figure S4h. Field dependence of the magnetization for **Coapy** at 1.9 K from -30000 Oe to 70000 Oe.

Figure S4i. Field dependence of the magnetization for **Cobpee** at 1.9 K from -20000 Oe to 20000 Oe.

Figure S4j. Field dependence of the magnetization for **Cobpy** at temperatures ranging from 1.9 K to 10.0 K in the 0-70000 and 0-5000 ranges.

Figure S4k. Field dependence of the magnetization for **Coapy** at temperatures ranging from 1.9 K to 10.0 K in the 0-70000 range and temperatures ranging from 1.9 K to 3.0 K in the 0-5000 range.

Figure S4l. Field dependence of the magnetization for **Cobpee** at temperatures ranging from 1.9 K to 10.0 K in the 0-70000 range and temperatures ranging from 1.9 K to 2.8 K in the 0-5000 range.

Figure S4m. Temperature dependence of the in-phase and out-of phase *ac* susceptibility of **Cobpy** at different frequencies between 1 to 1500 Hz with an *ac* field amplitude of 3 Oe in zero *dc* field.

Figure S4n. Temperature dependence of the in-phase and out-of phase *ac* susceptibility of **Coapy** at different frequencies between 1 to 1500 Hz with an *ac* field amplitude of 3 Oe in zero dc field.

Figure S4o. Temperature dependence of the in-phase and out-of phase *ac* susceptibility of **Cobpee** at different frequencies between 1 to 1500 Hz with an *ac* field amplitude of 3 Oe in zero dc field.

S1. Infrared spectra

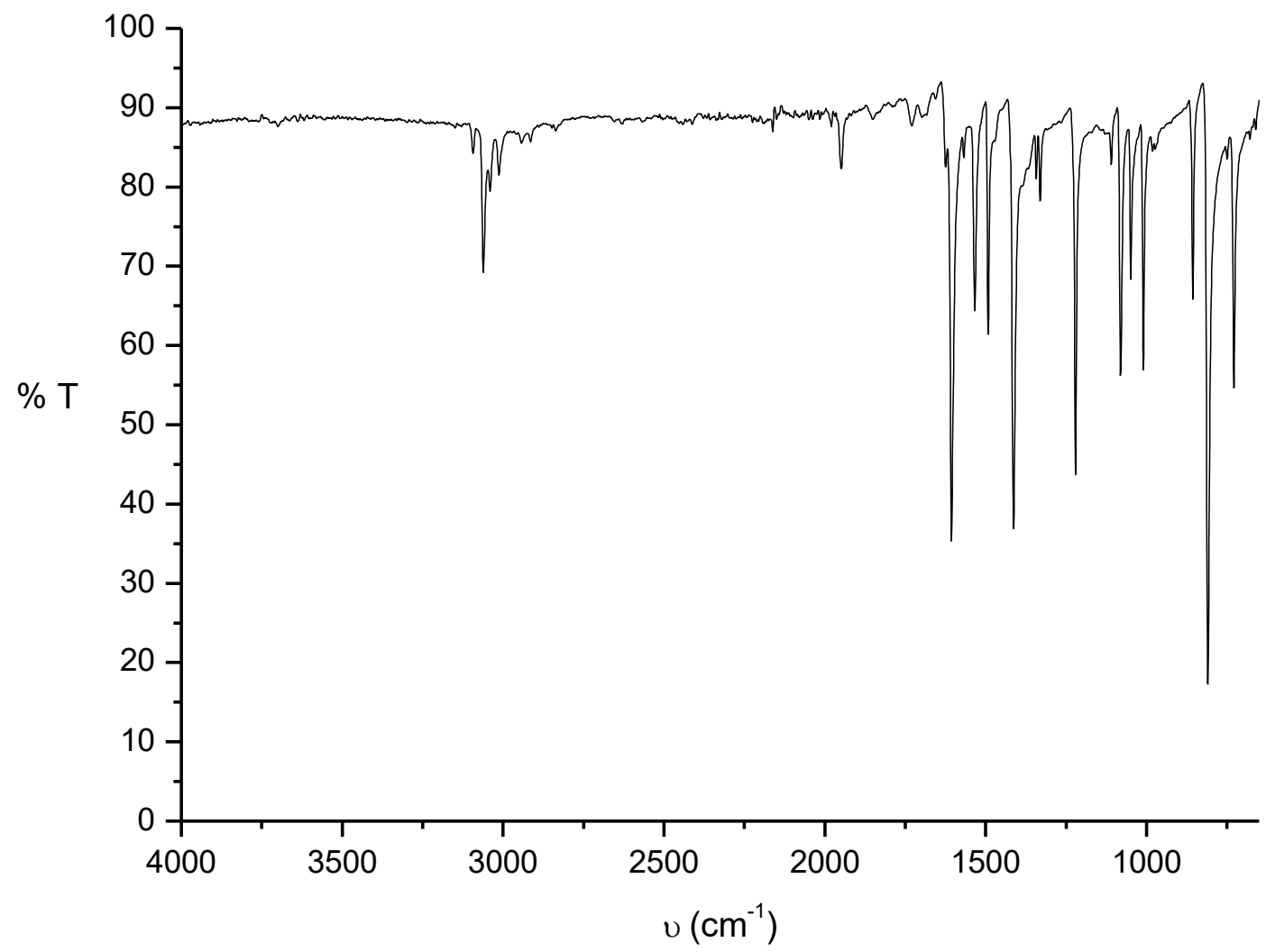

Figure S1a. IR spectrum of $[CoCl_2(N,N')]_n$ [*N*,*N*' = 4,4'-bipyridine (4,4'-bpy), (**Cobpy**). Absorptions: 3061w, 1948w, 1606s, 1534m, 1492m, 1413s, 1220s, 1081m, 1049m, 1010m, 856m, 809s, 728s.

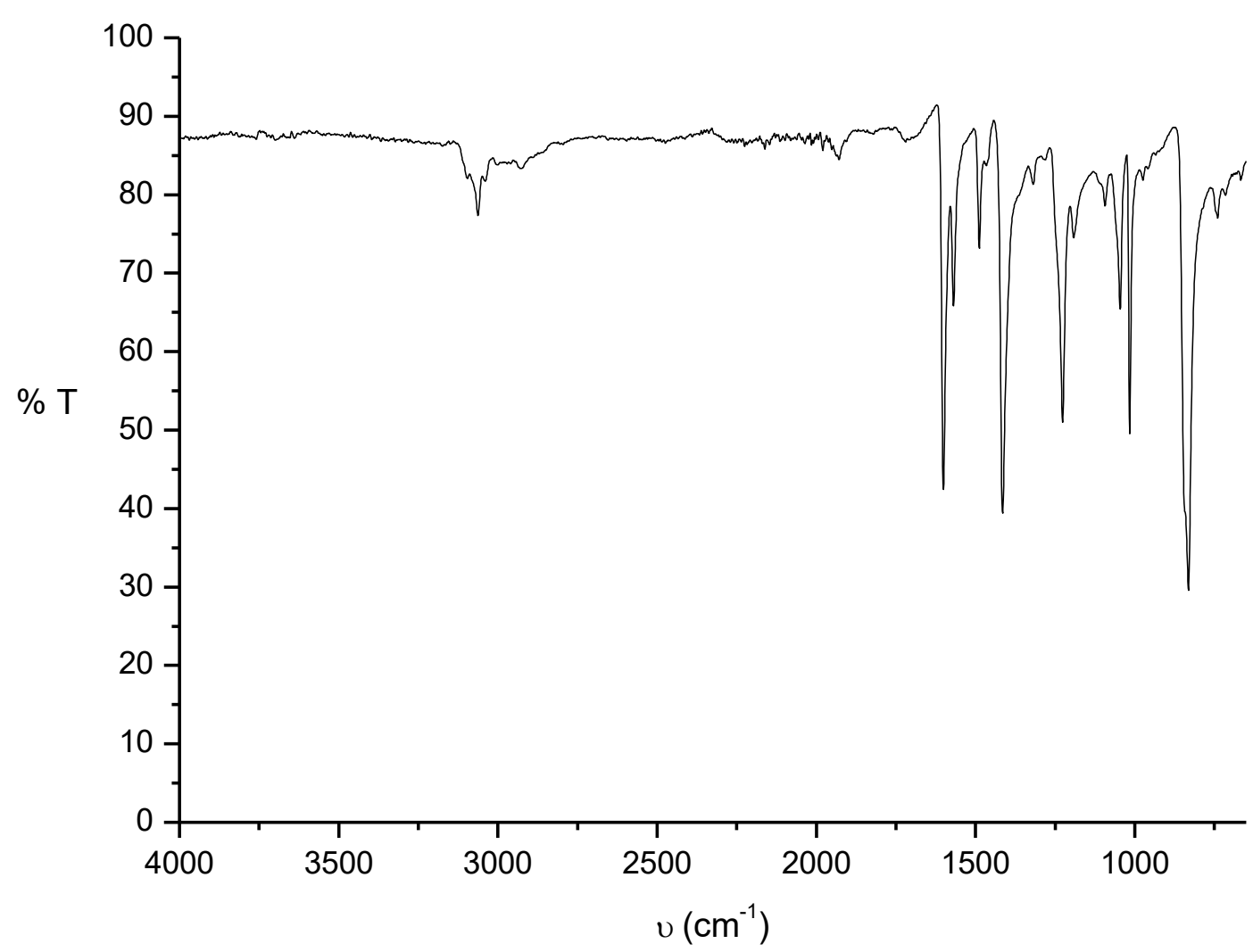

Figure S1b. IR spectrum of $[CoCl_2(N,N')]_n$ [*N*,*N*' = *trans*-4,4'-azopyridine (*t*-apy), (**Coapy**). Absorptions: 3062w, 1928w, 1601s, 1569m, 1488m, 1415s, 1227s, 1191m, 1046m, 1016s, 829s, 739w.

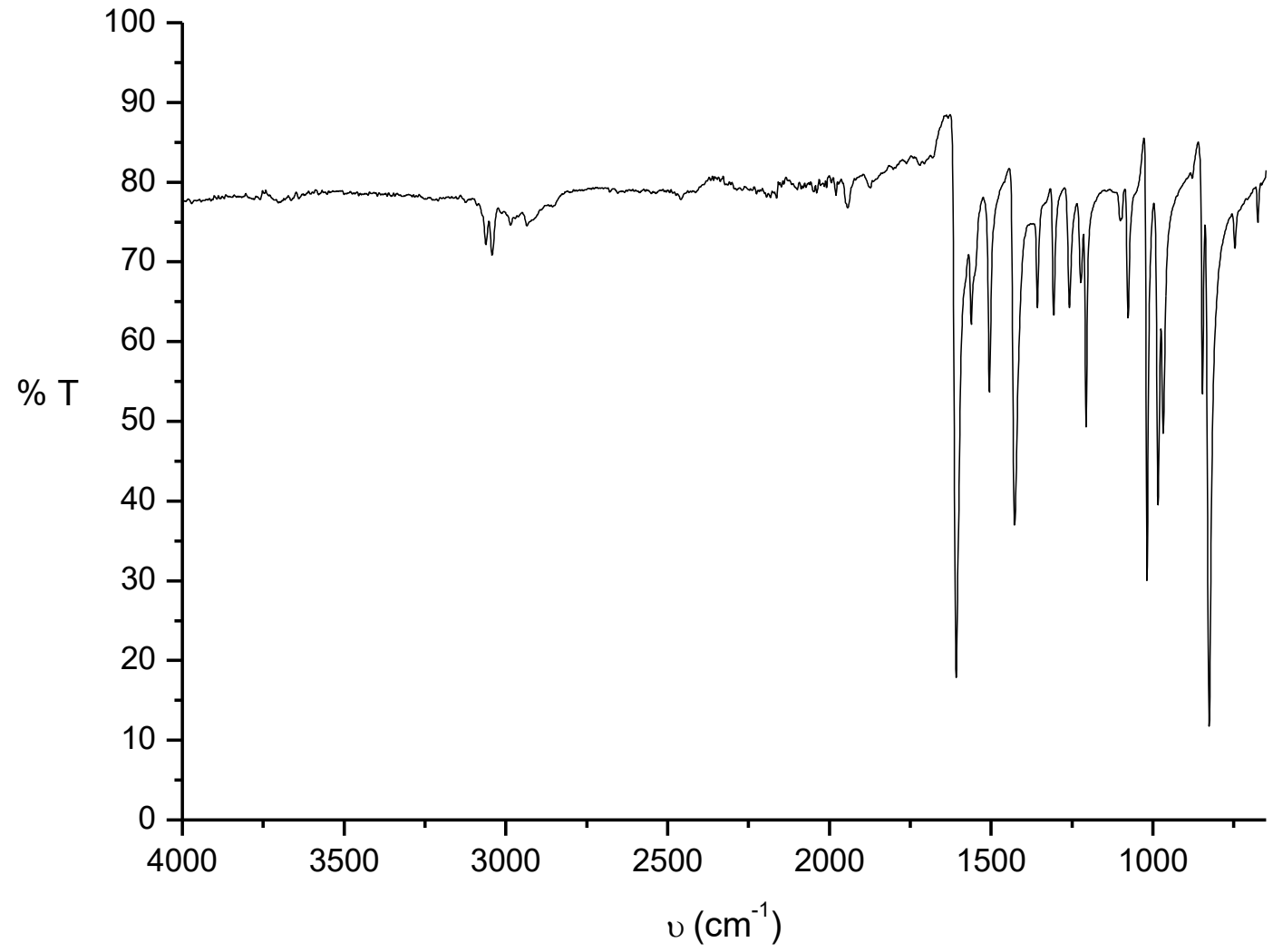

Figure S1c. IR spectrum of $[CoCl_2(N,N')]_n$ [*N*,*N*' = *trans*-1,2-bis(4-pyridyl)ethylene (*t*-bpee), (**Cobpee**). Absorptions: 3061w, 3042w, 1608s, 1561m, 1505m, 1428s, 1357m, 1307m, 1257m, 1207m, 1077m, 1018s, 984s, 968s, 847m, 827s.

S2. Thermogravimetric analysis

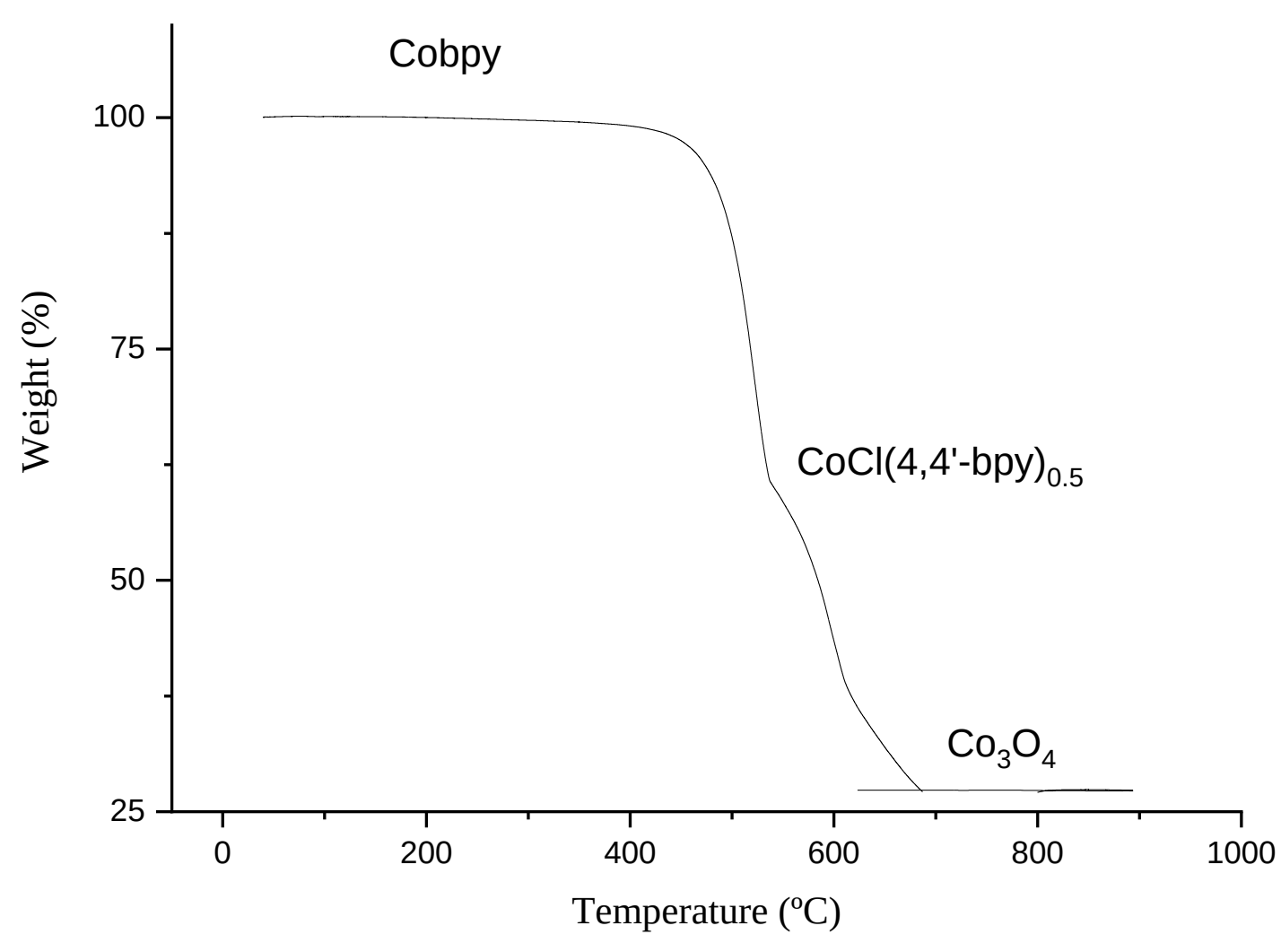

Figure S2a. Thermogravimetric curve for **Cobpy** and tentative assignments of the formed species. Air atmosphere, 5 °C·min^{-1}.

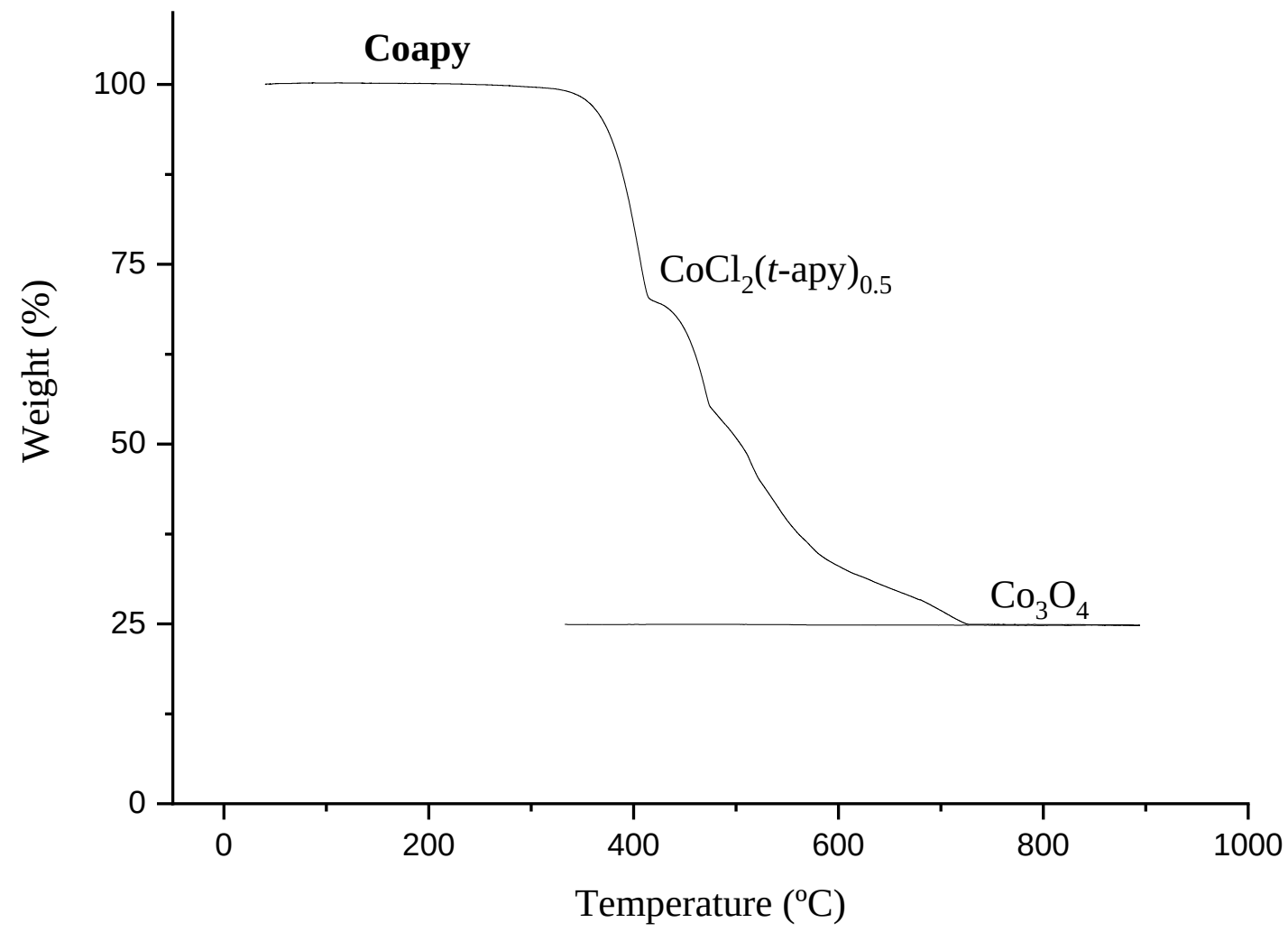

Figure S2b. Thermogravimetric curve for **Coapy** and tentative assignments of the formed species. Air atmosphere, 5 °C·min^{-1}.

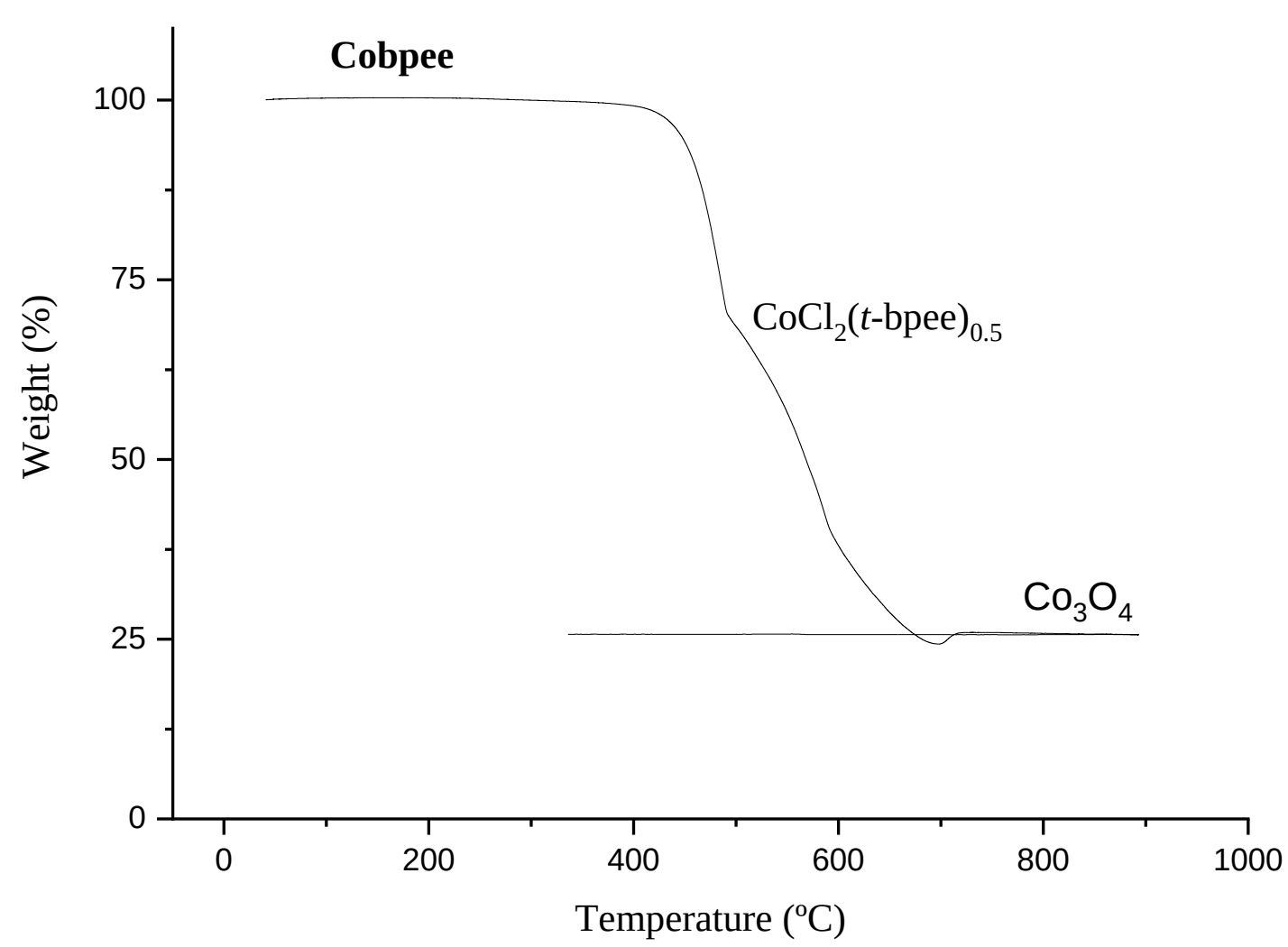

Figure S2c. Thermogravimetric curve for **Cobpee** and tentative assignments of the formed species. Air atmosphere, 5 °C·min^{-1}.

Table S2a. Weight loss observed for **Cobpy**, **Coapy** and **Cobpee** in their thermogravimetric curves and tentative assignments of the formed species.

Compound	Weight loss *T* (ºC)	Product formed	Theoretical loss (%)	Experimental loss (%)
**Cobpy**	460	CoCl(4,4'-bpy)$_{0.5}$	39.3	39.7
	535	Co_3O_4	71.9	72.5
**Coapy**	350	$CoCl_2$(*t*-apy)$_{0.5}$	29.3	29.5
	430	Co_3O_4	74.3	75.3
**Cobpee**	425	$CoCl_2$(*t*-bpee)$_{0.5}$	29.3	29.8
	490	Co_3O_4	74.3	74.8

S3. Powder X ray diffraction of the compounds

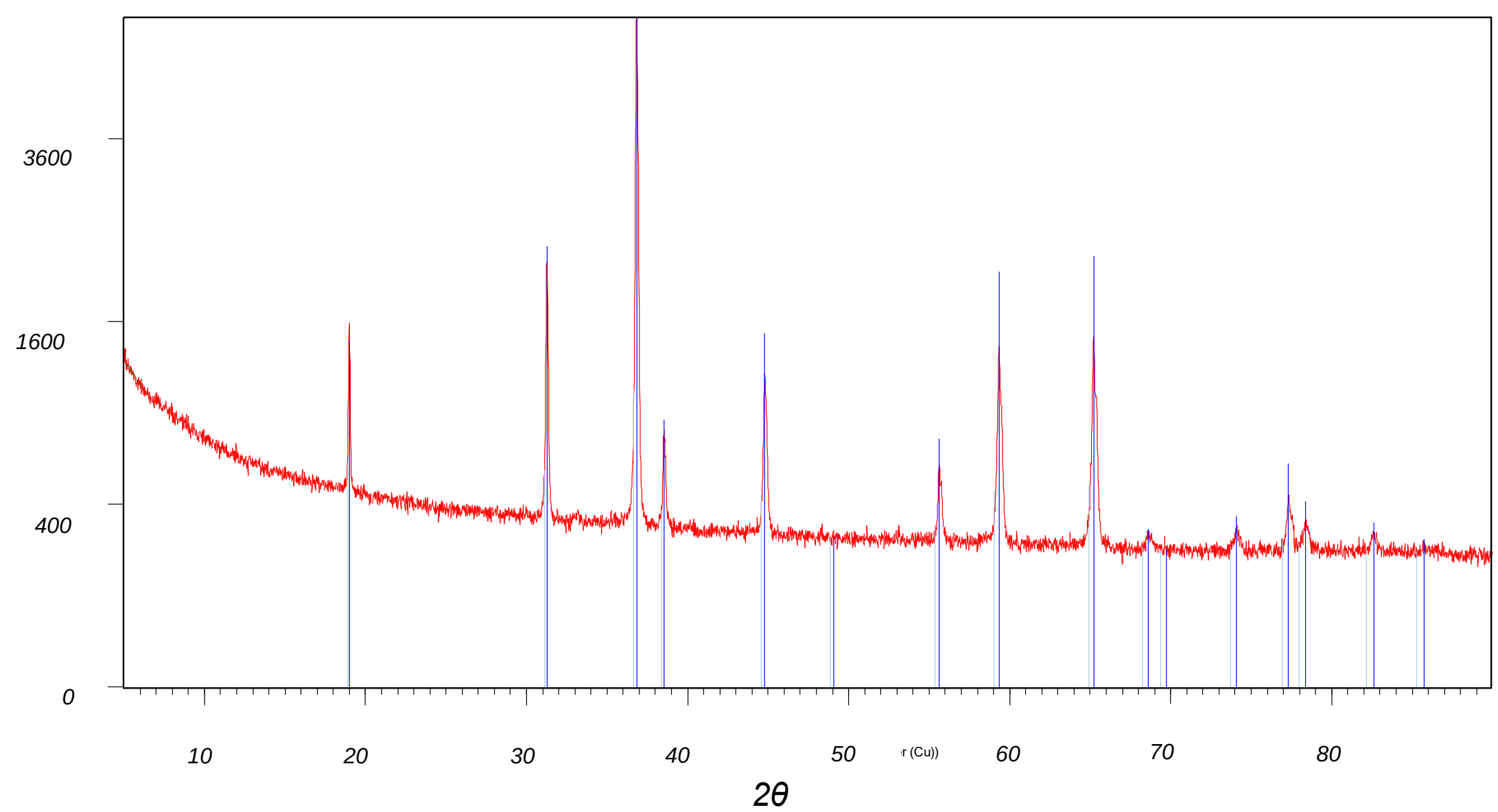

Figure S3a. Powder X-ray diffraction data for Co_3O_4 obtained after the thermogravimetric measurement of **Cobpy** in air.

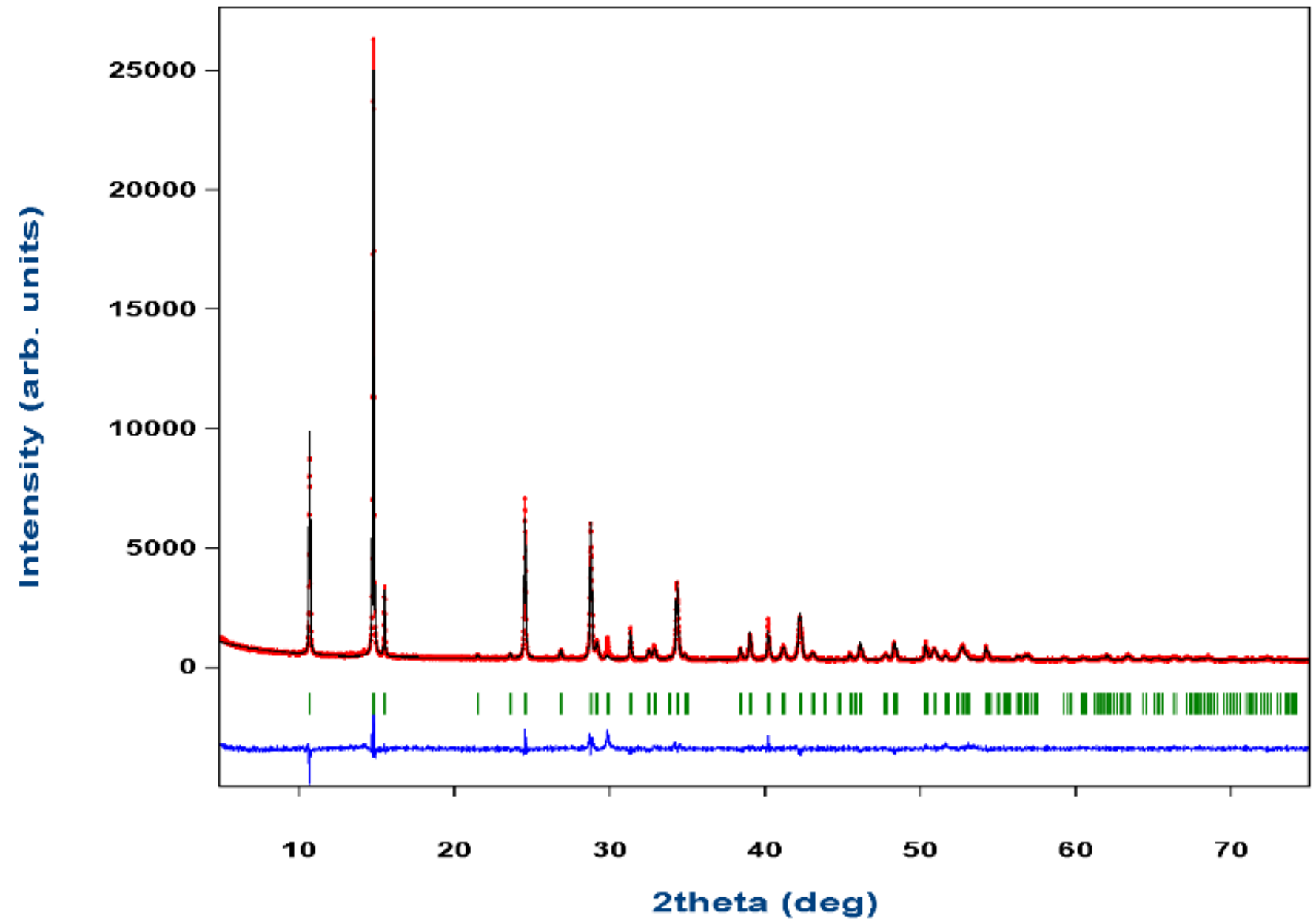

Figure S3b. Rietveld refinement plot for **Cobpy**. Red dots represent experimental XRD scan whereas the black line represents the computed scan for QEJCOX CSD entry. Green tics are the peak positions for the refined phase. Blue line shows the difference between the observed and calculated patterns. Agreement factors: R_p= 6.15, R_{wp}= 9.68, R_{Bragg}= 7.81 and Chi^2= 3.16.

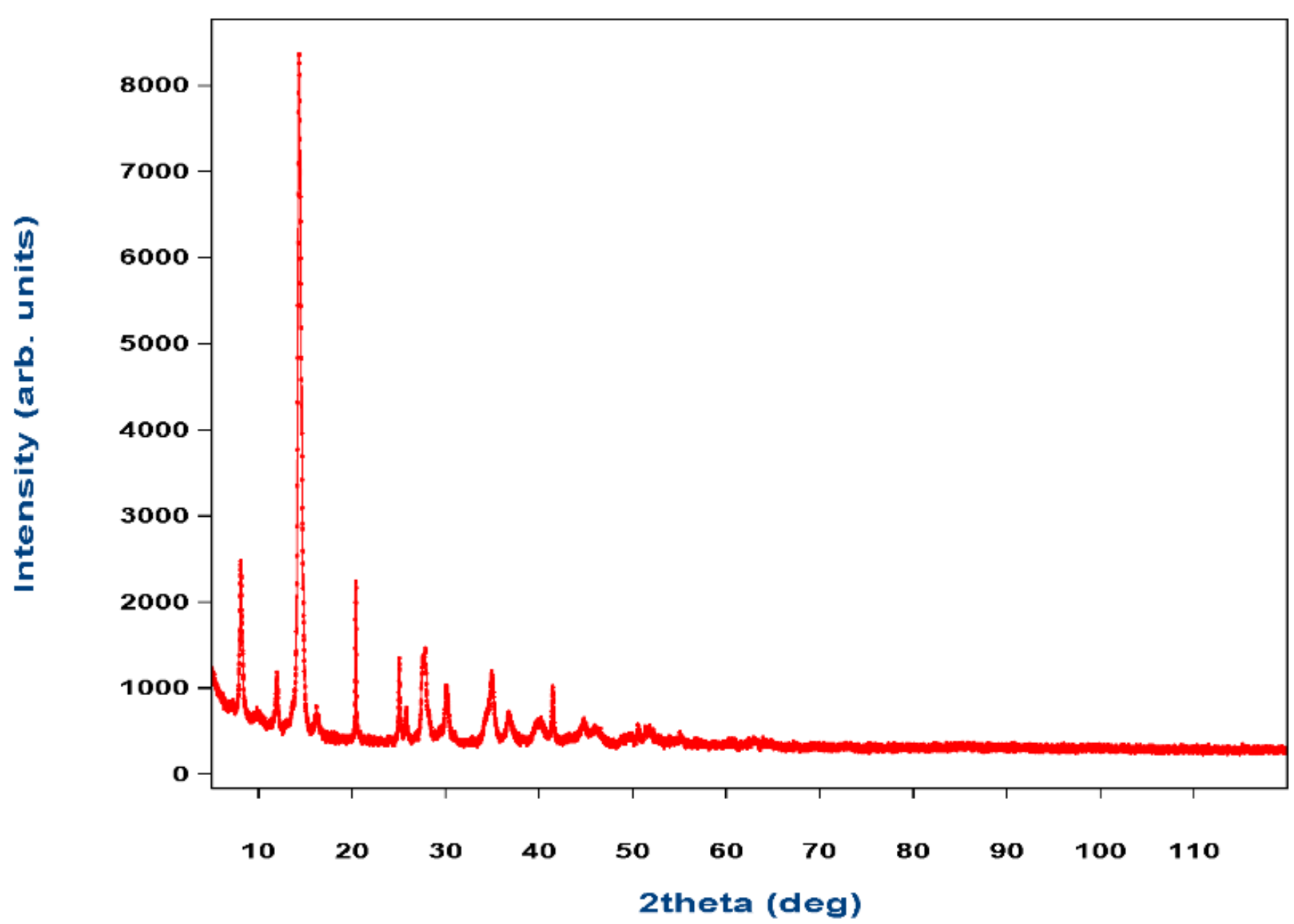

Figure S3c. X-ray powder diffraction scan for **Coapy**.

S4. Magnetic measurements

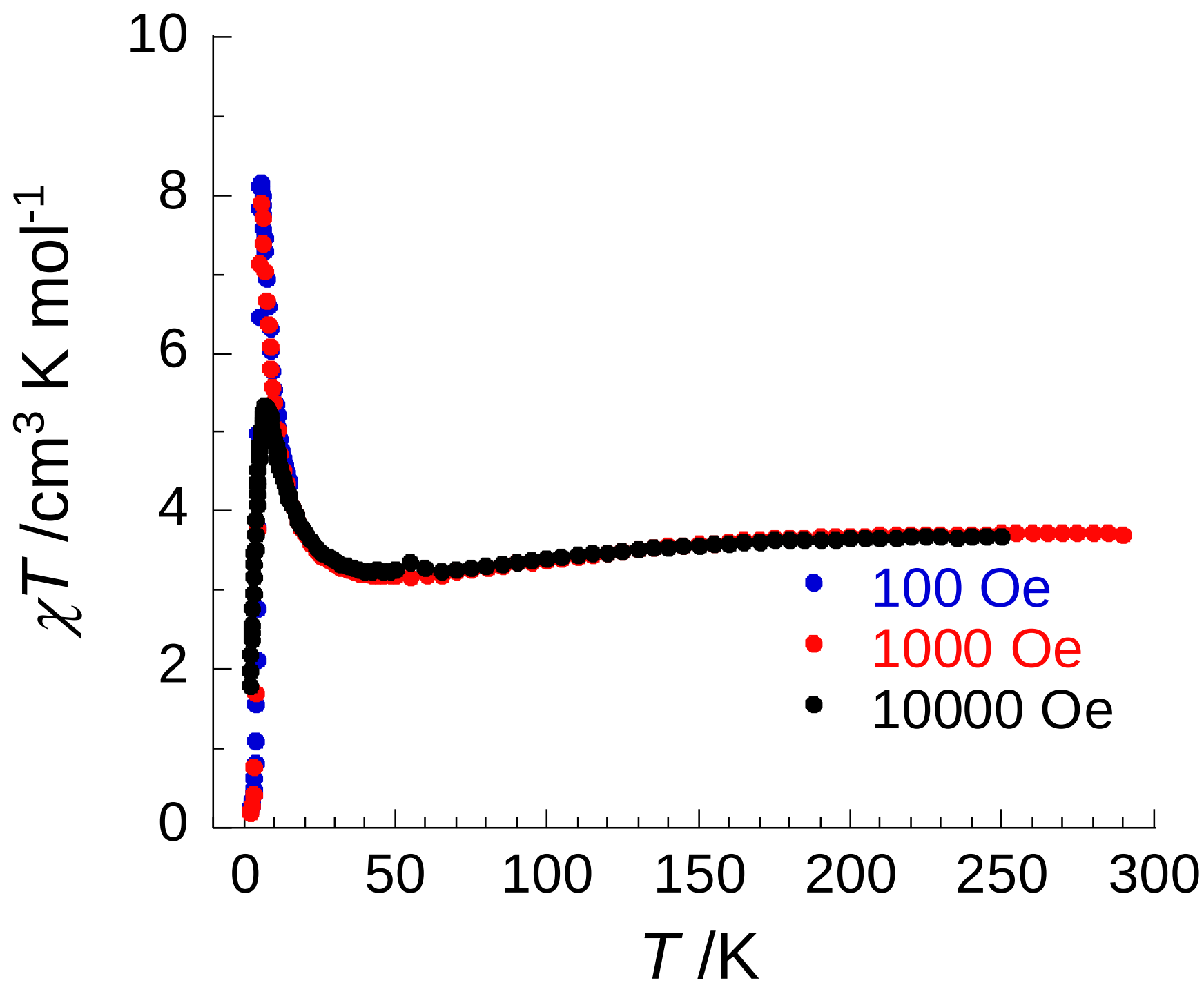

Figure S4a. Temperature dependence of χT for **Cobpy** at 100, 1000 and 10000 Oe.

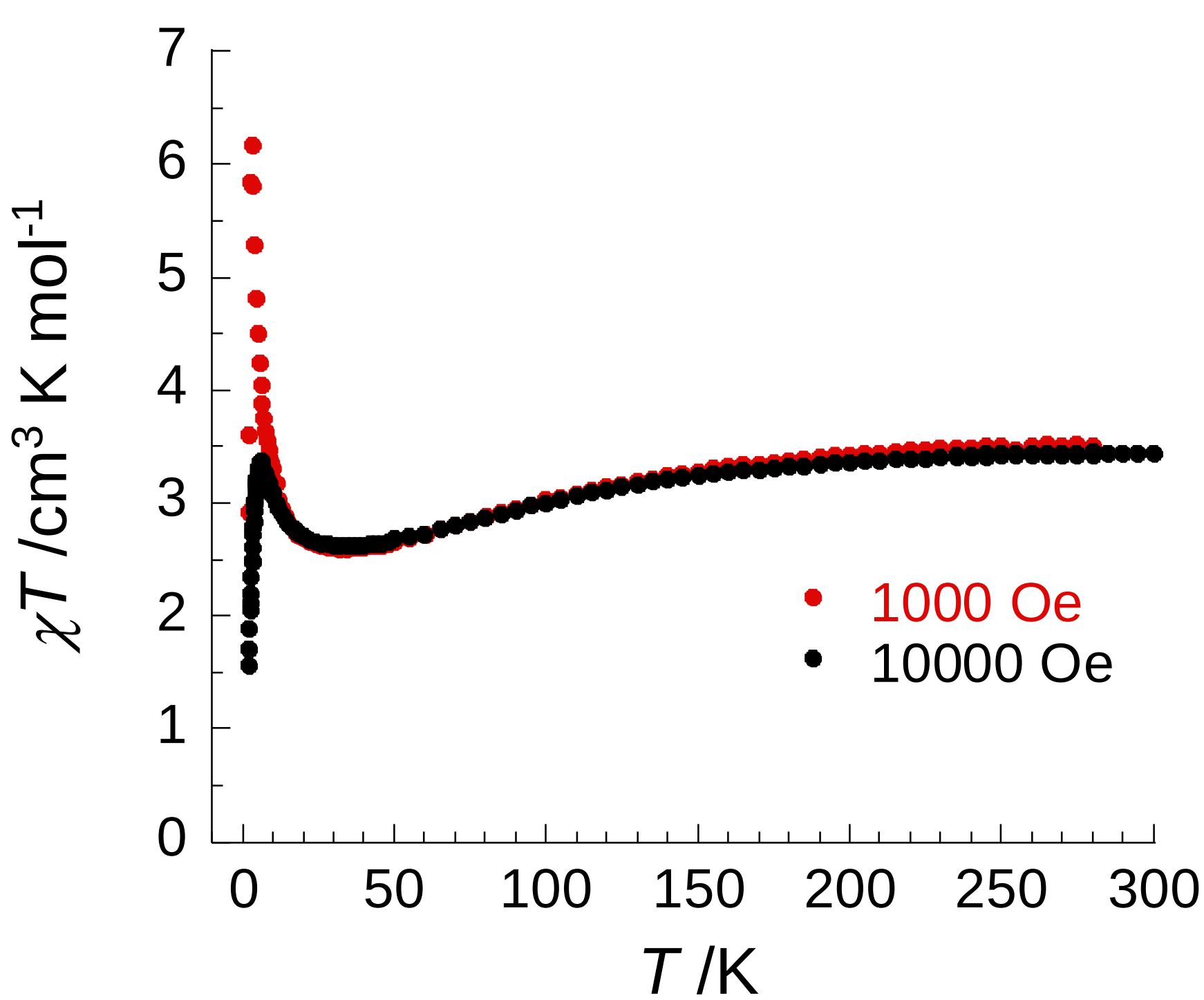

Figure S4b. Temperature dependence of χT for **Coapy** at 1000 and 10000 Oe.

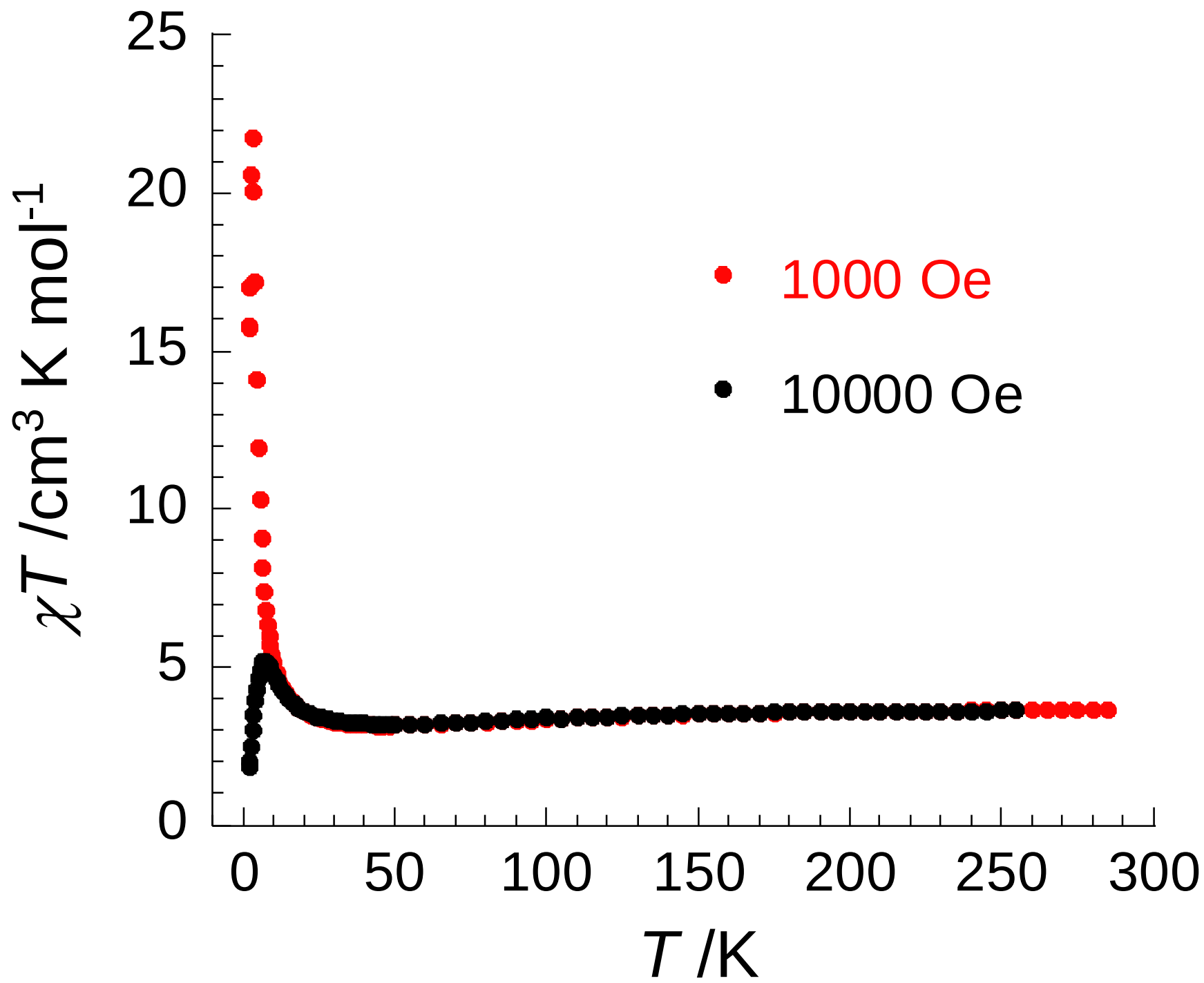

Figure S4c. Temperature dependence of χT for **Cobpee** at 1000 and 10000 Oe.

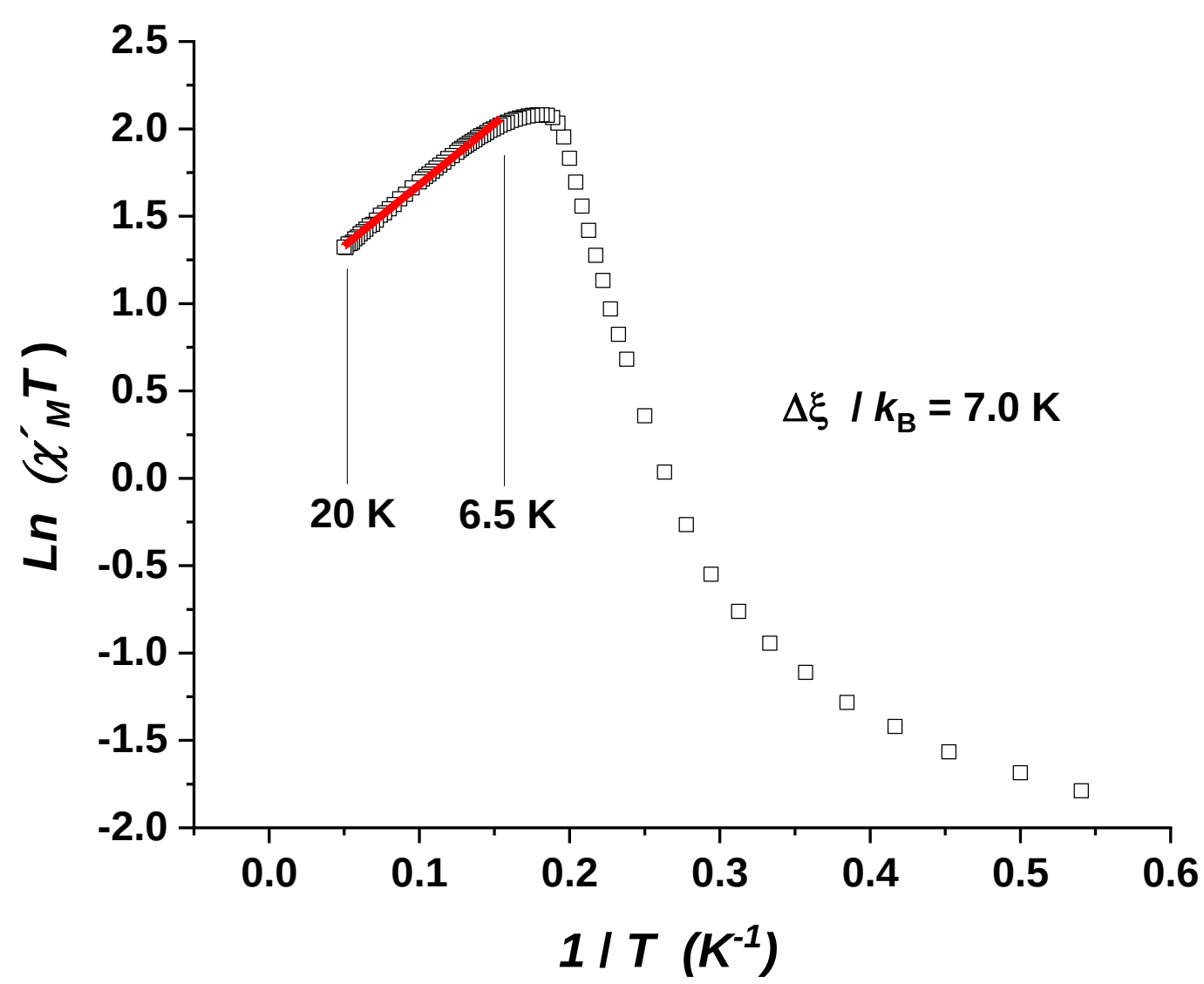

Figure S4d. Ln $\chi'T$ *vs* 1/*T* plot of **Cobpy** (where χ' is the in-phase *ac* molar susceptibility at 1 Hz in zero *dc* field). The solid line corresponds to the fit of the linear portion of the data between 6.5 and 20 K.

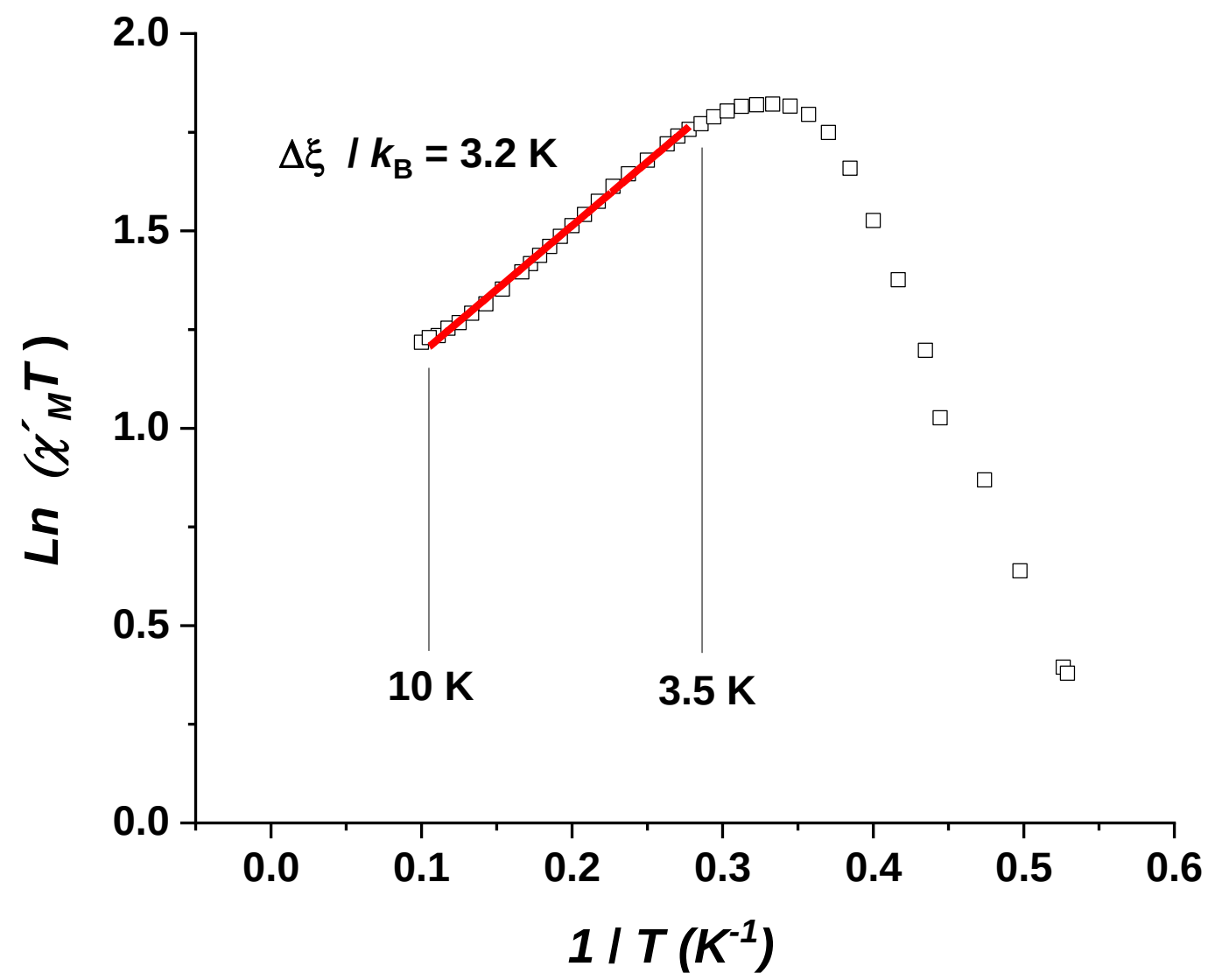

Figure S4e. Ln $\chi' T$ *vs* 1/*T* plot of **Coapy** (where χ' is the in-phase *ac* molar susceptibility at 1 Hz in zero *dc* field). The solid line corresponds to the fit of the linear portion of the data between 3.5 and 10 K.

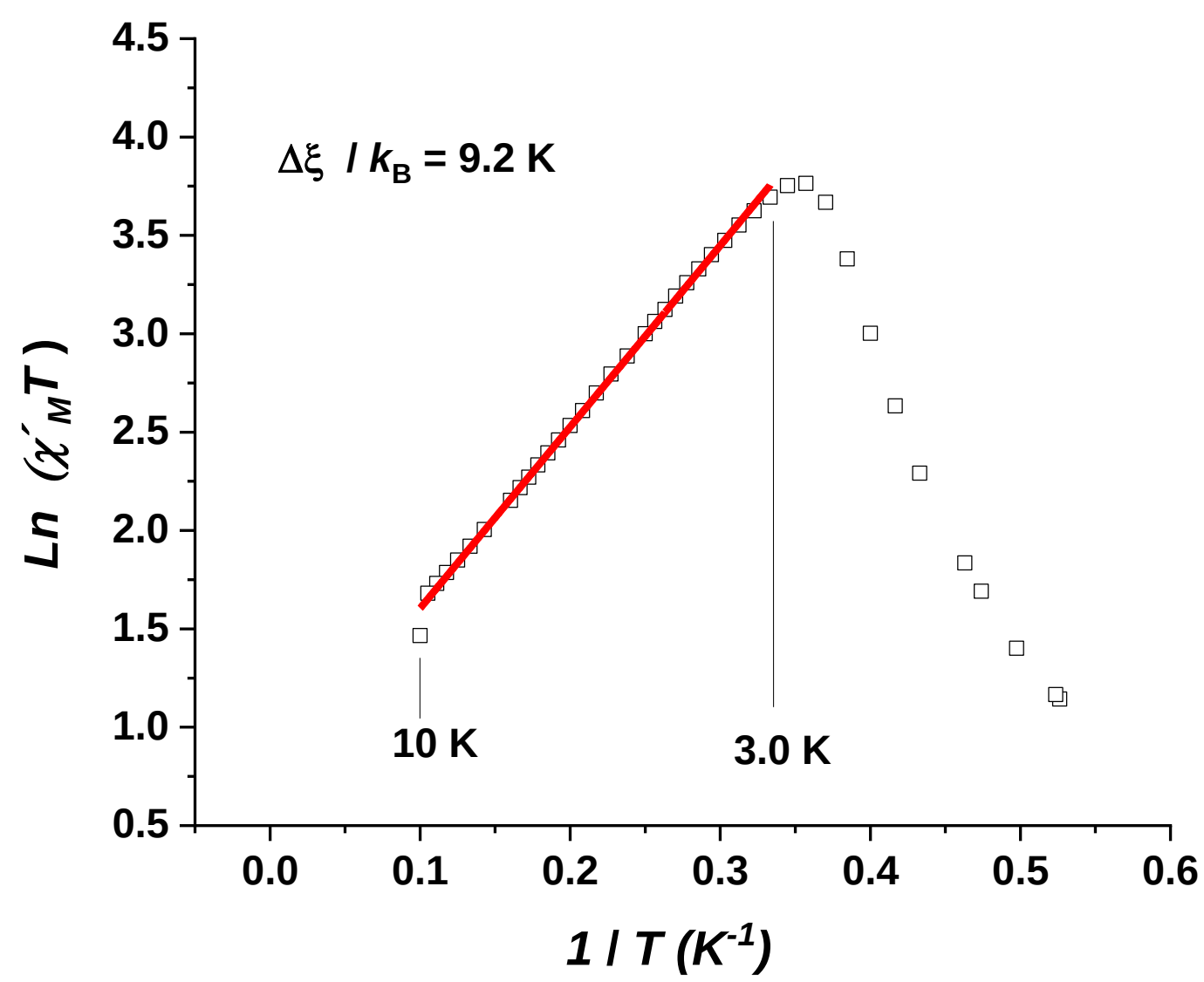

Figure S4f. Ln $\chi' T$ *vs* 1/*T* plot of **Cobpee** (where χ' is the in-phase *ac* molar susceptibility at 1 Hz in zero *dc* field). The solid line corresponds to the fit of the linear portion of the data between 10 and 3 K.

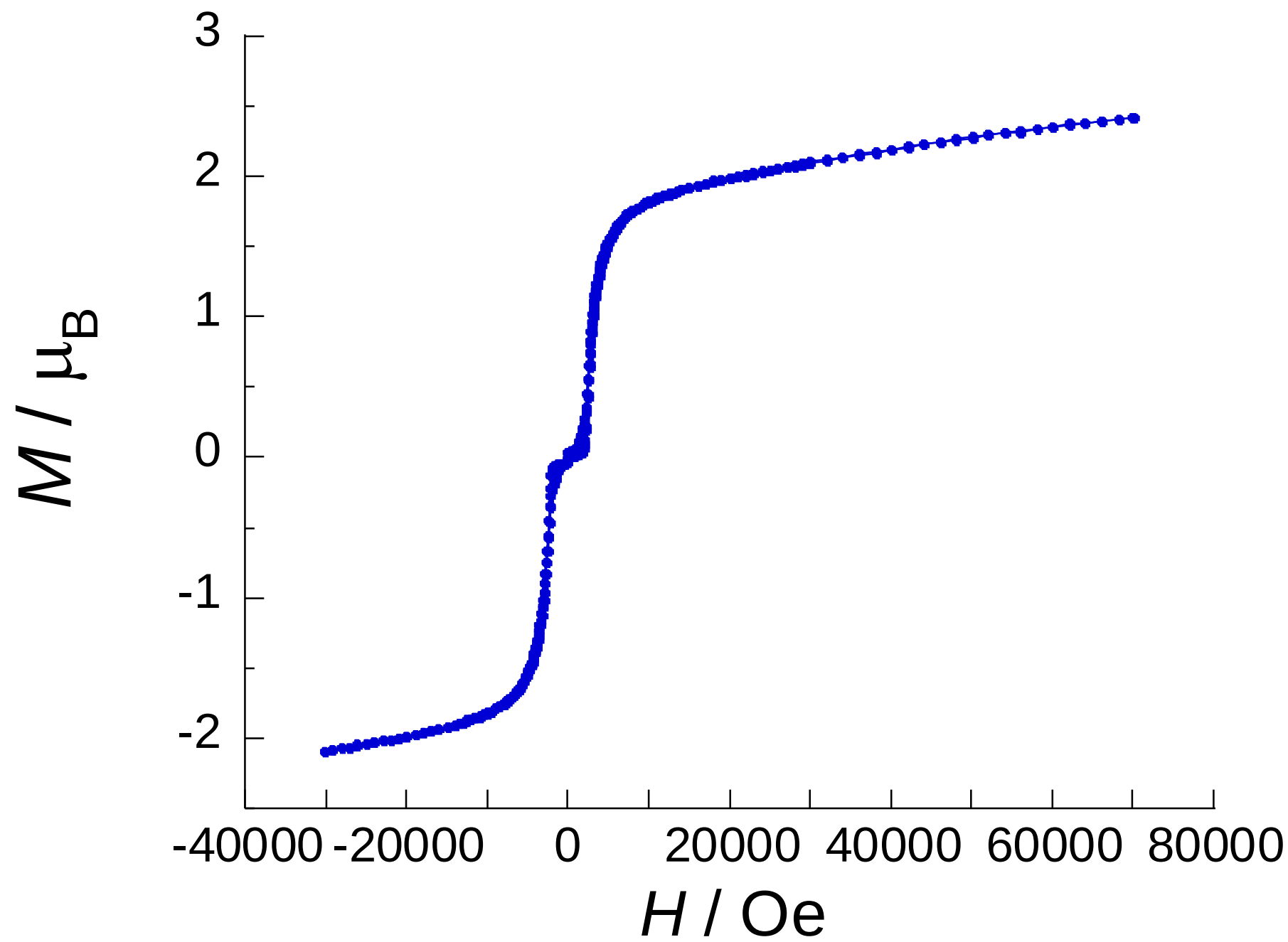

Figure S4g. Field dependence of the magnetization for **Cobpy** at 1.9 K from -30000 Oe to 70000 Oe. Solid lines are just a guide for the eye.

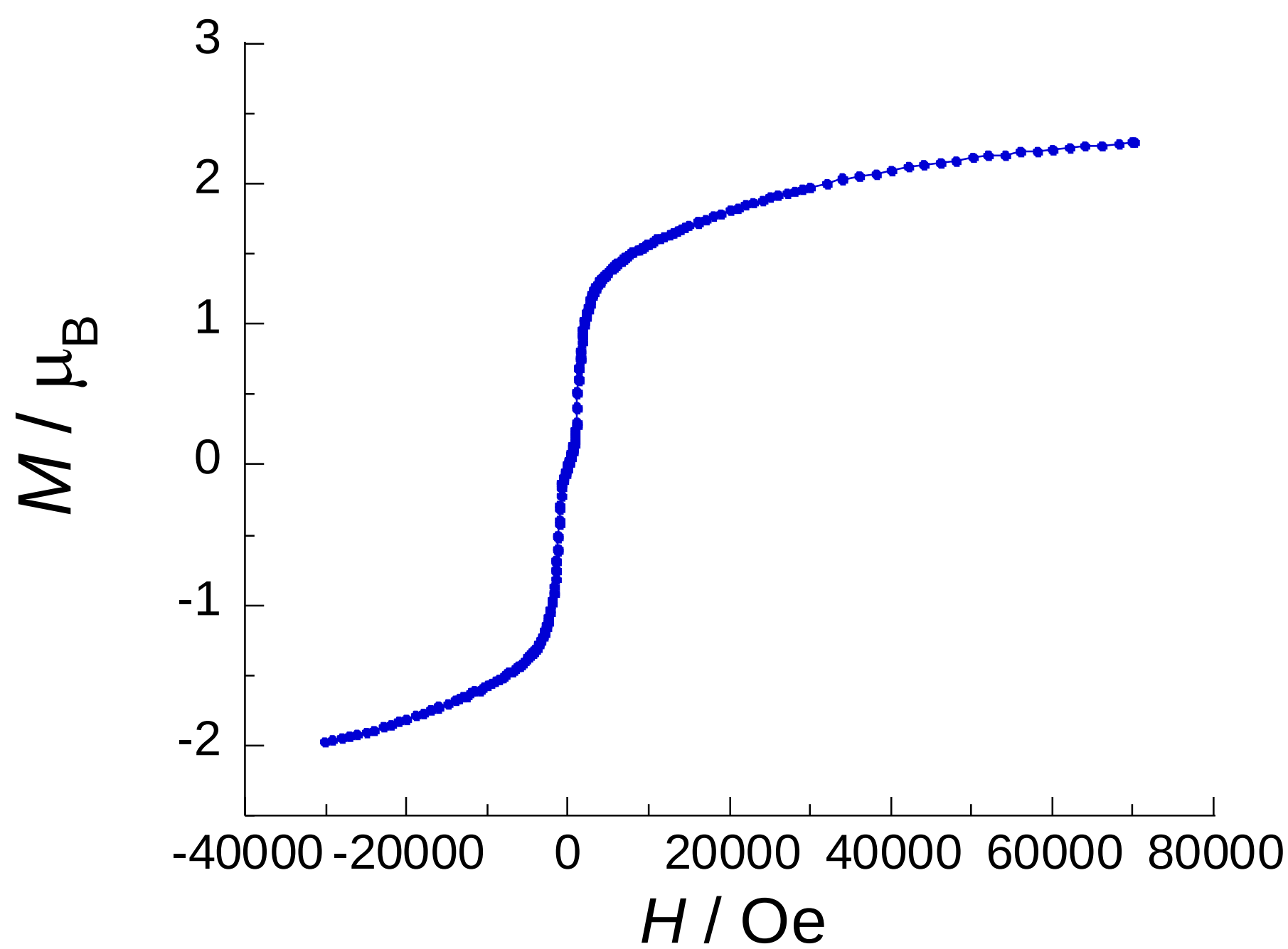

Figure S4h. Field dependence of the magnetization for **Coapy** at 1.9 K from -30000 Oe to 70000 Oe. Solid lines are just a guide for the eye.

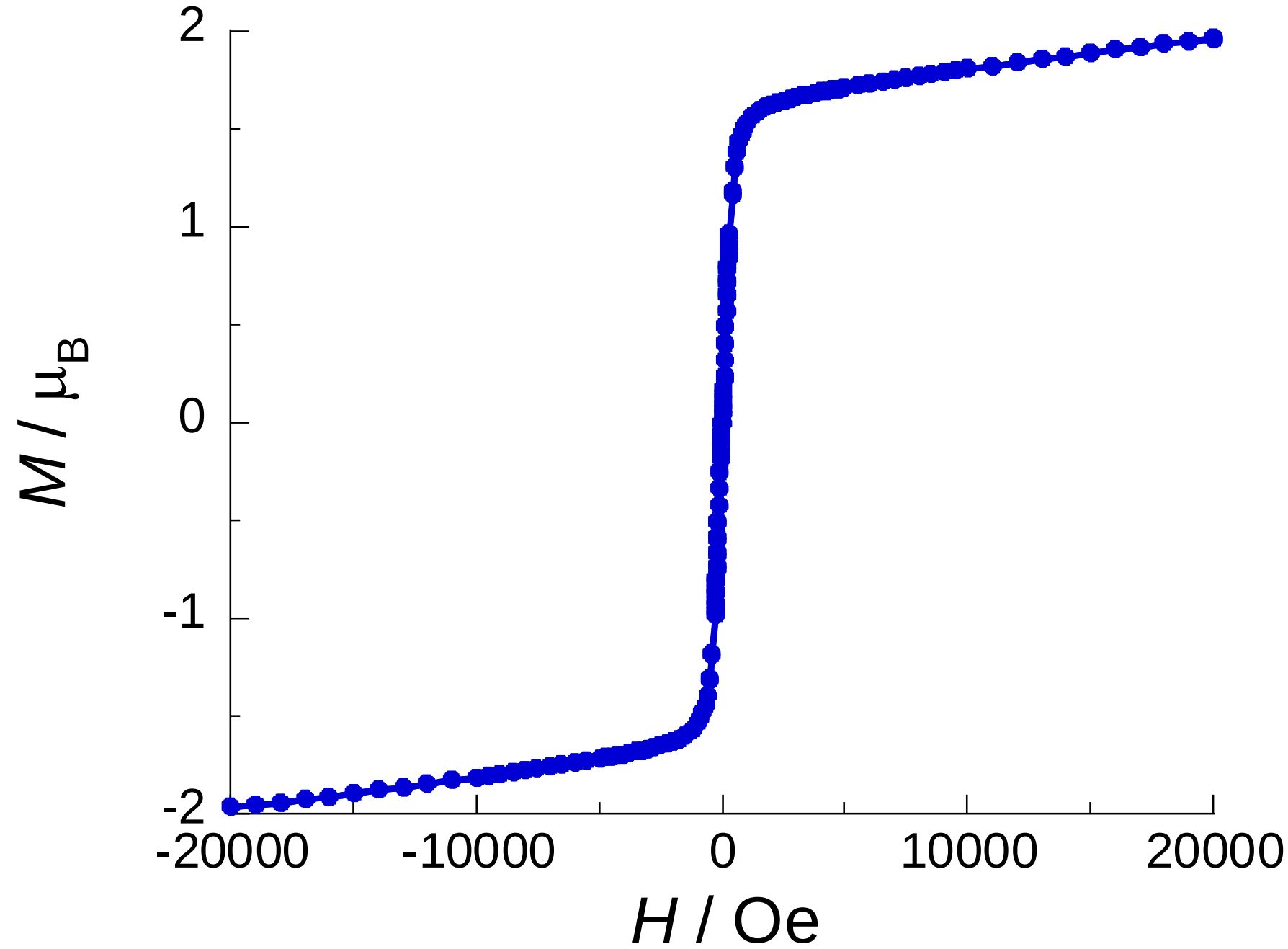

Figure S4i. Field dependence of the magnetization for **Cobpee** at 1.9 K from -20000 Oe to 20000 Oe. Solid lines are just a guide for the eye.

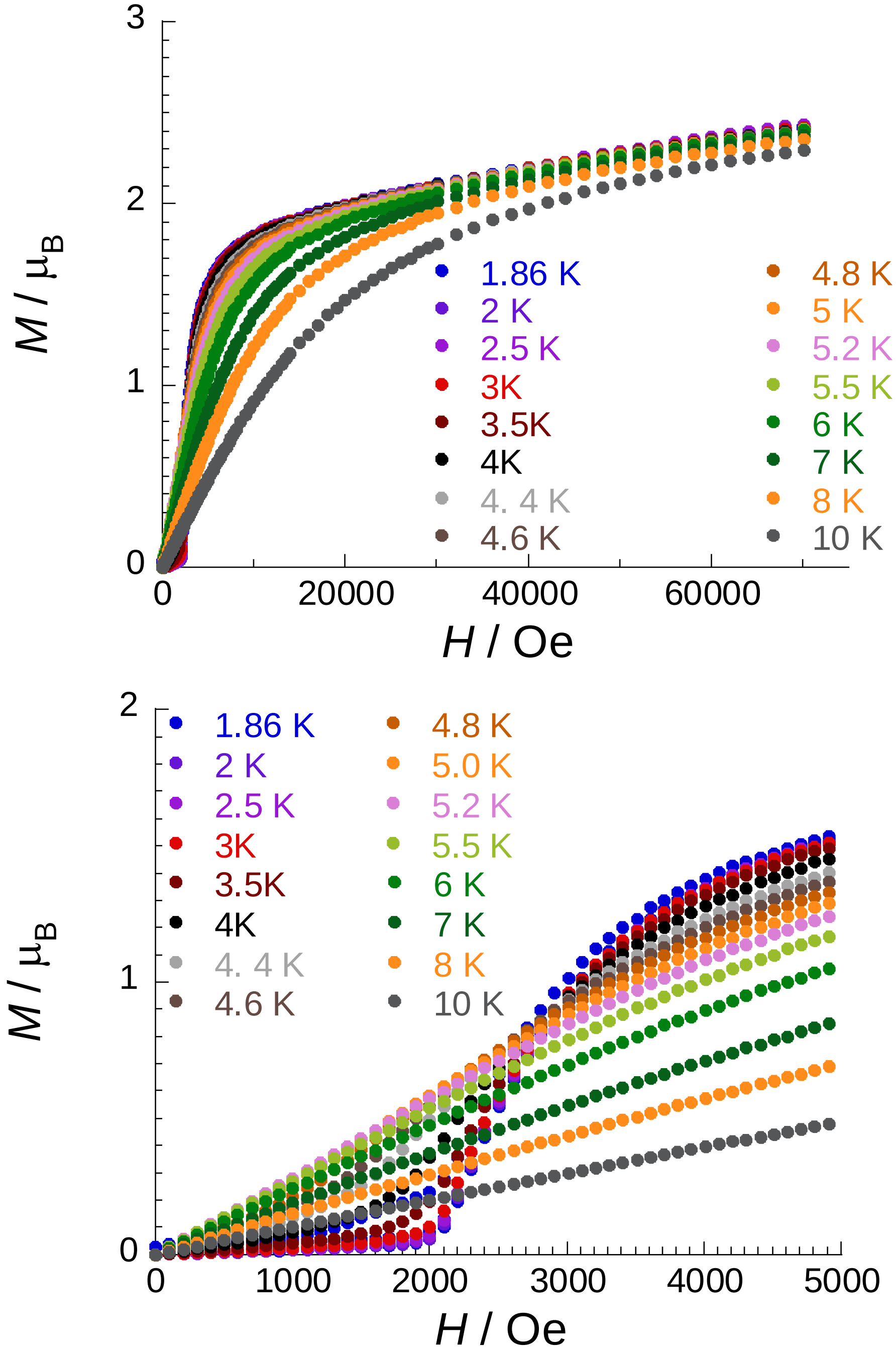

Figure S4j. Field dependence of the magnetization for **Cobpy** at temperatures ranging from 1.9 K to 10 K in the 0-70000 (top) and 0-5000 (bottom) ranges.

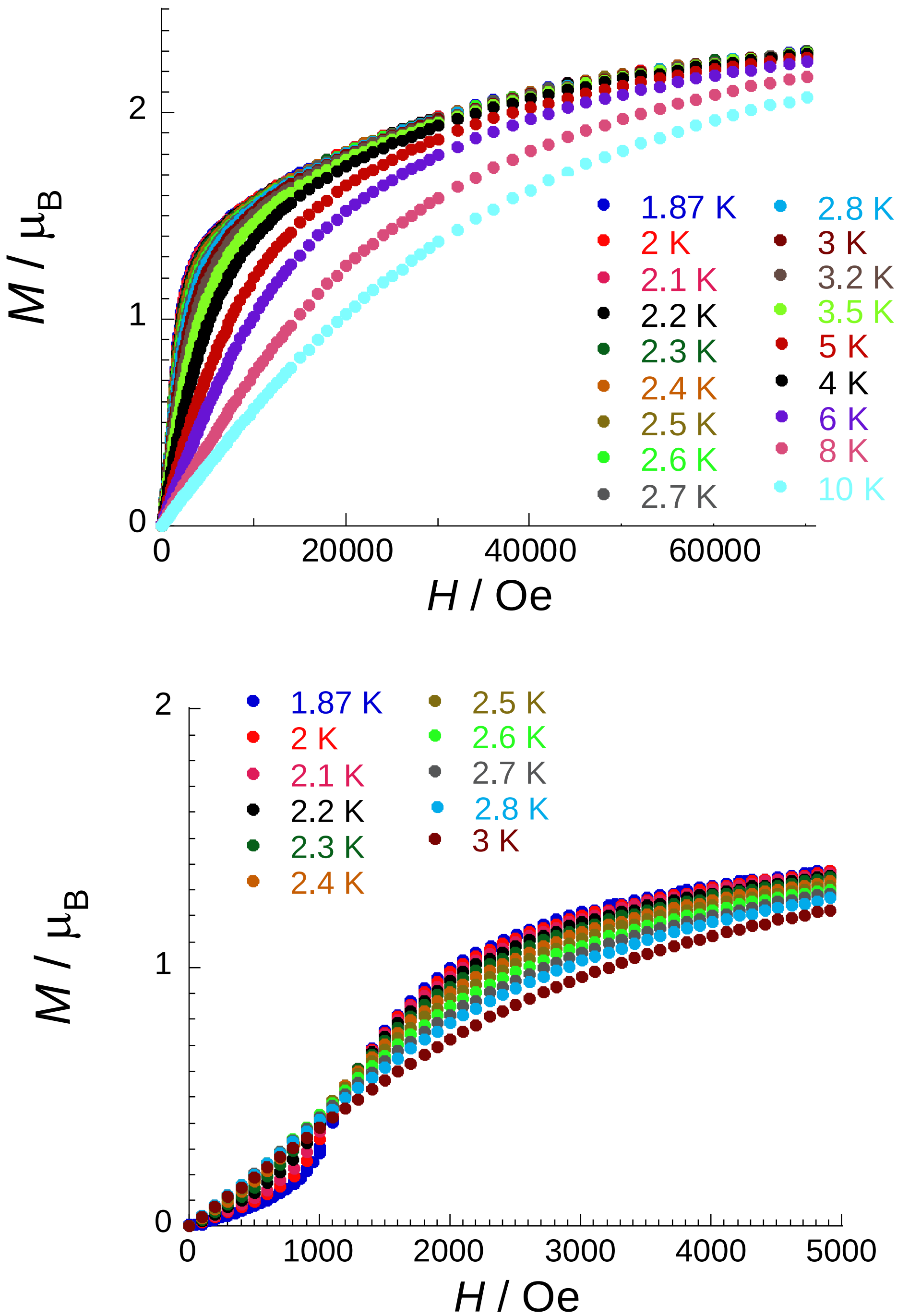

Figure S4k. Field dependence of the magnetization for **Coapy** at temperatures ranging from 1.9 K to 10.0 K in the 0-70000 range (top) and temperatures ranging from 1.9 K to 3.0 K in the 0-5000 range (bottom).

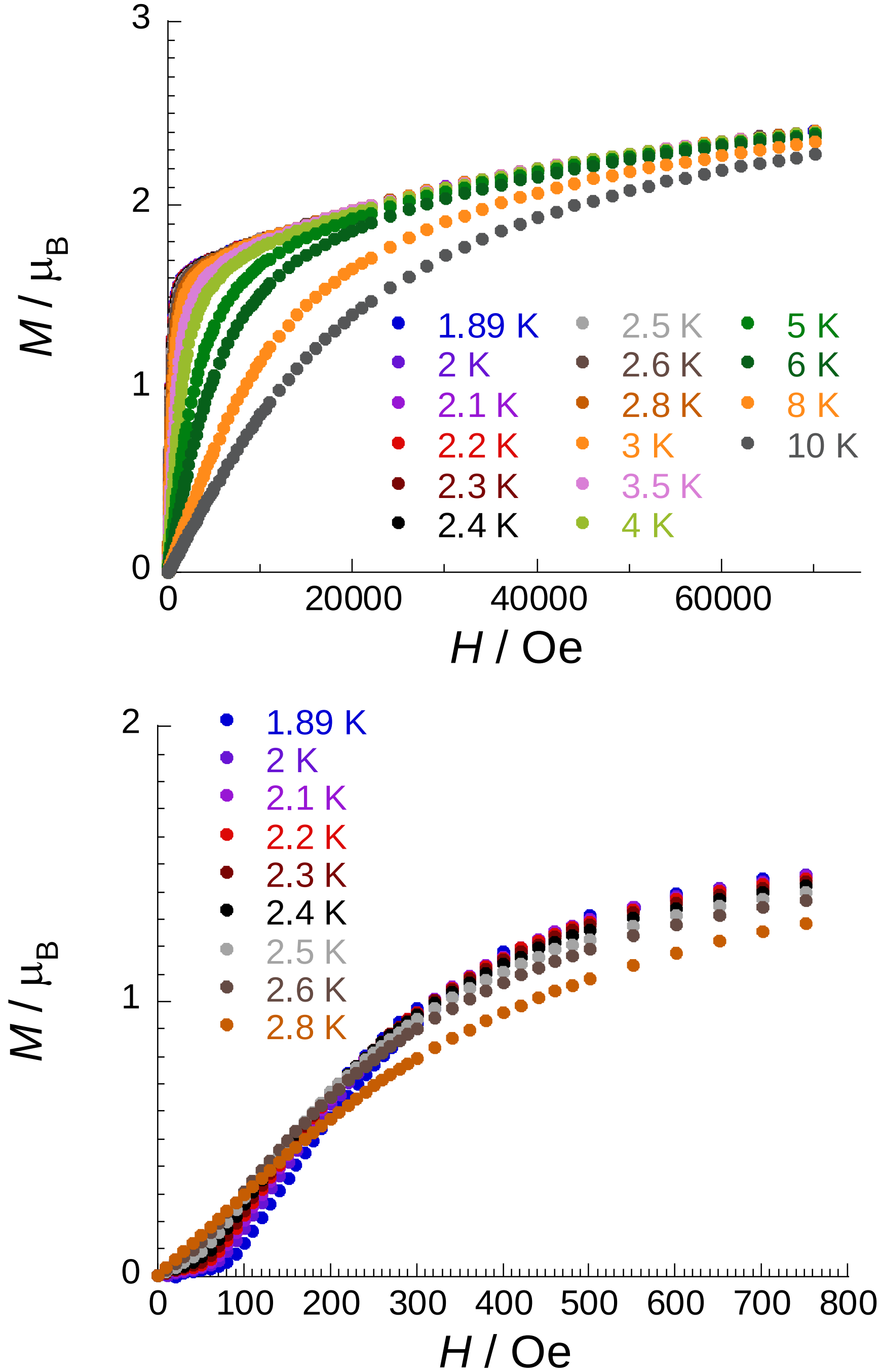

Figure S4l. Field dependence of the magnetization for **Cobpee** at temperatures ranging from 1.9 K to 10.0 K in the 0-70000 range (top) and temperatures ranging from 1.9 K to 2.8 K in the 0-5000 range (bottom).

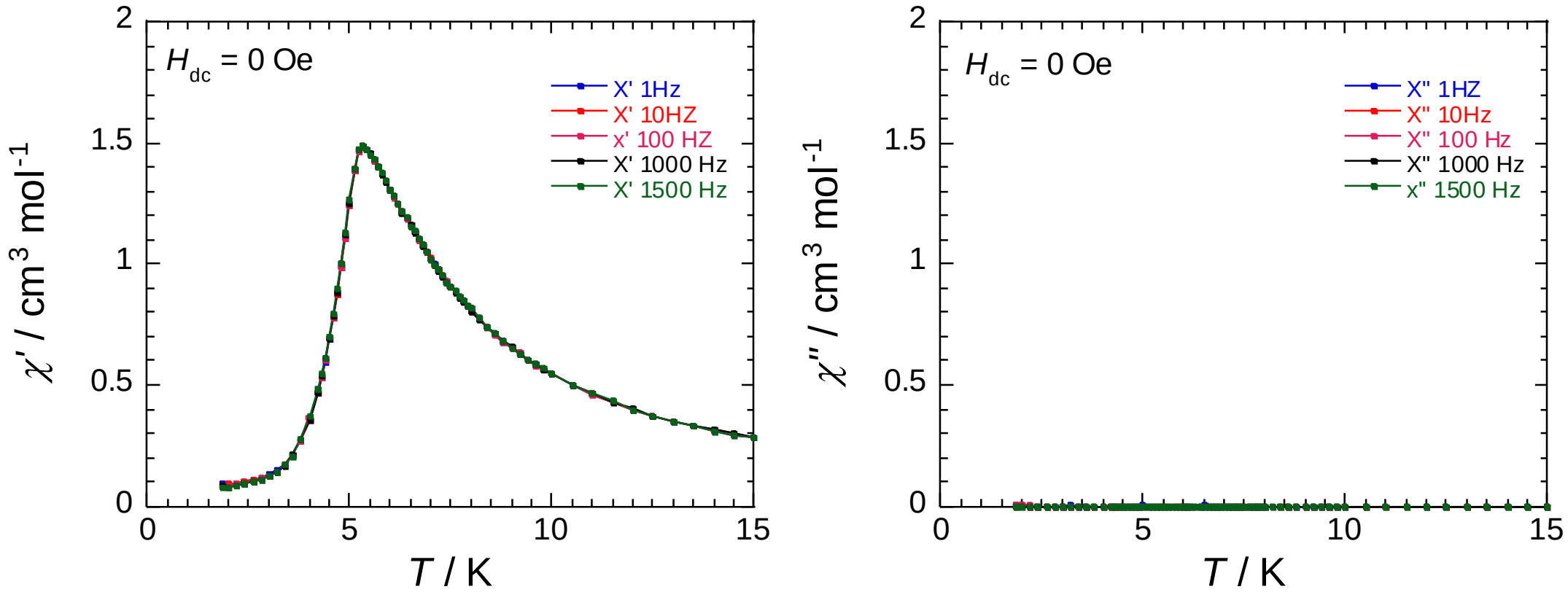

Figure S4m. Temperature dependence of the in-phase (left) and out-of phase (right) *ac* susceptibility of **Cobpy** at different frequencies between 1 to 1500 Hz with an *ac* field amplitude of 3 Oe in zero *dc* field.

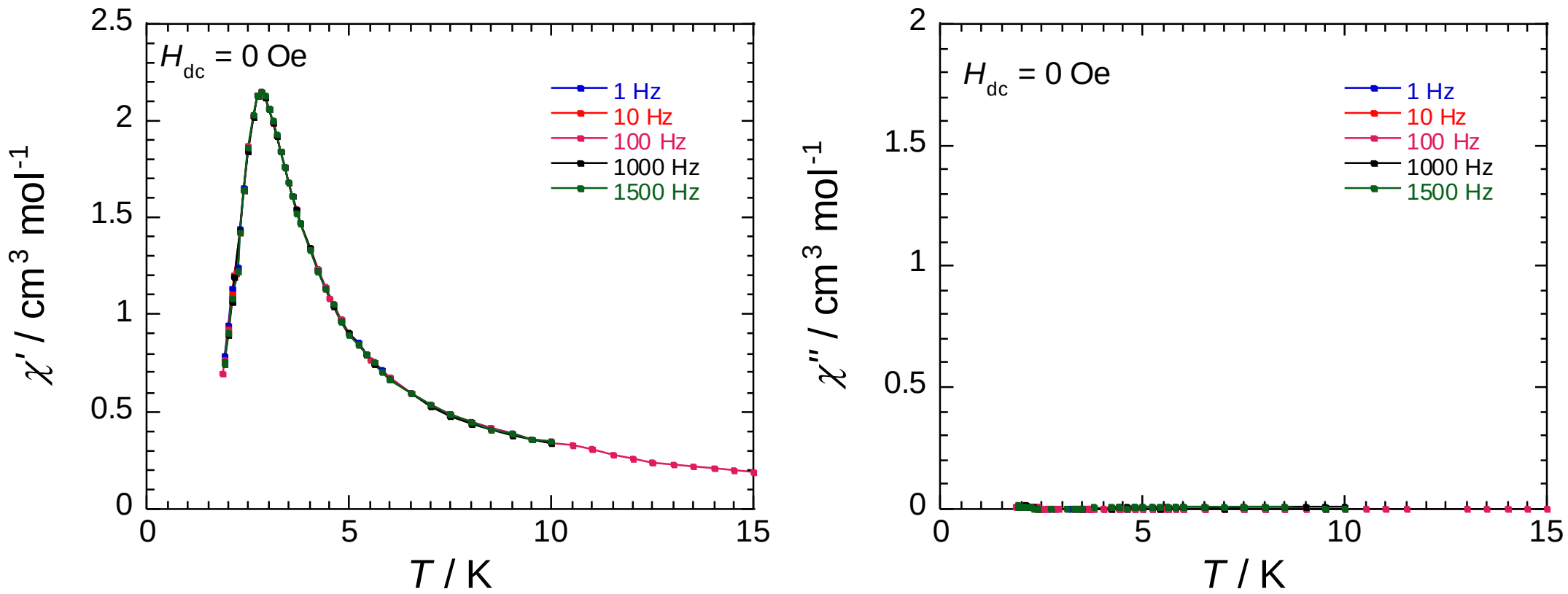

Figure S4n. Temperature dependence of the in-phase (left) and out-of phase (right) *ac* susceptibility of **Coapy** at different frequencies between 1 to 1500 Hz with an *ac* field amplitude of 3 Oe in zero *dc* field.

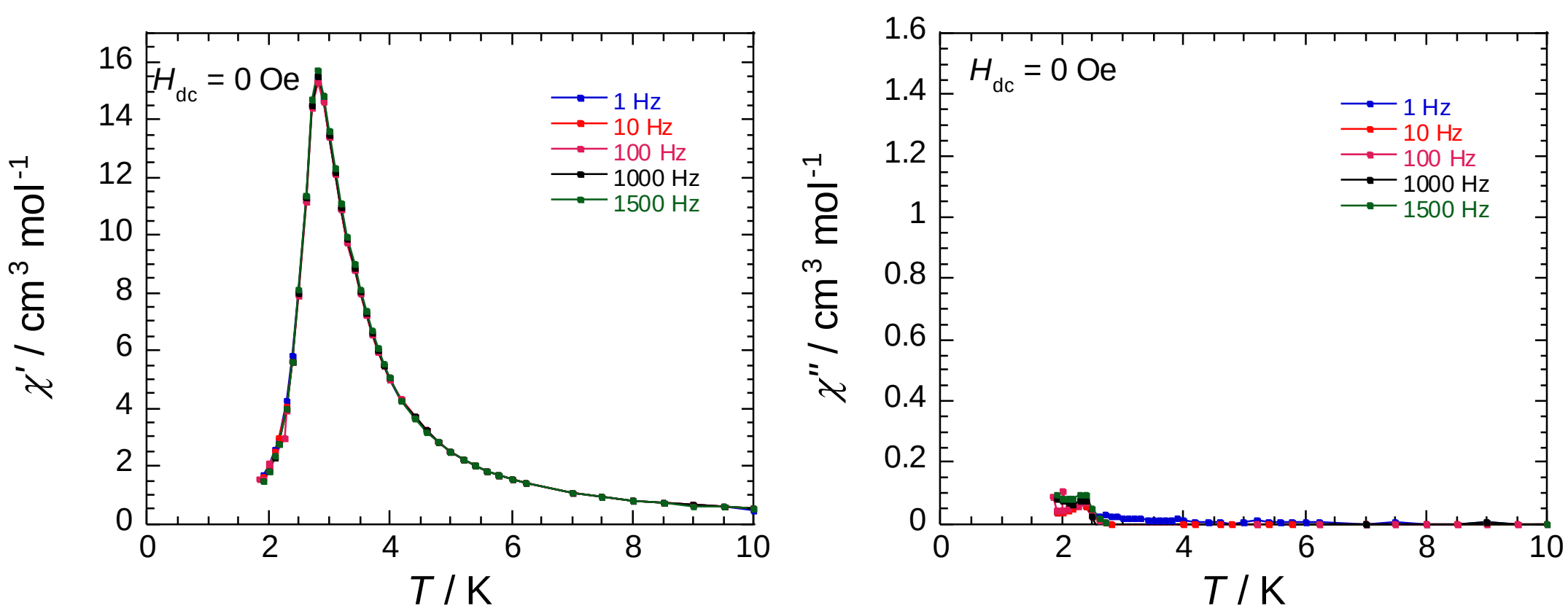

Figure S4o. Temperature dependence of the in-phase (left) and out-of phase (right) *ac* susceptibility of **Cobpee** at different frequencies between 1 to 1500 Hz with an *ac* field amplitude of 3 Oe in zero *dc* field.
